# A Systematic Literature Review of You Only Look Once Architectures (v1–v12) in Healthcare Systems

**DOI:** 10.3390/diagnostics16060935

**Published:** 2026-03-22

**Authors:** Ozgur Koray Sahingoz, Gozde Karatas Baydogmus, Emin Kugu

**Affiliations:** 1 Computer Engineering Department, Biruni University, 34015 Istanbul, Türkiye; 2 Department of Computer Science, Loyola University Chicago, Chicago, IL 60660, USA; gkaratasbaydogmus@luc.edu; 3 Software Engineering Department, TED University, 06420 Ankara, Türkiye; emin.kugu@tedu.edu.tr

**Keywords:** object detection, medical imaging, health informatics, YOLO, You Only Look Once

## Abstract

**Background/Objectives**: The integration of deep learning and computer vision into healthcare has improved medical diagnosis and image analysis. Among object detection algorithms, the YOLO family has attracted substantial attention due to its ability to analyze images in real time with reported improvements in detection performance across multiple studies. This systematic review examines the evolution of YOLO algorithms for diagnostic applications in healthcare from YOLOv1 to YOLOv12. **Methods**: Peer-reviewed scientific articles published up to 1 January 2026 were retrieved from major scientific databases in accordance with PRISMA 2020 guidelines. The included studies applied YOLO models to medical imaging tasks, including disease and lesion detection and support for clinical procedures. Performance was synthesized using reported metrics such as average precision, accuracy, inference time, and computational efficiency. **Results**: The reviewed literature suggests progressive architectural refinements associated with reported improvements in diagnostic performance. YOLOv5 and YOLOv8 are the most frequently used architectures in diagnostic settings, reflecting a favorable trade-off between accuracy and computational complexity. YOLO-based methods have demonstrated strong performance across radiological, pathological, ophthalmological, and endoscopic applications. **Conclusions**: YOLO models have matured into robust and optimized solutions for medical image analysis; however, challenges remain in interpretability, cross-institution generalization, and deployment on edge devices. Future work on explainable YOLO-based diagnostics and energy-efficient model design will be particularly valuable.

## 1. Introduction

Object recognition refers to the process by which a computer or automated system identifies, categorizes, or labels objects within images or videos using visual features such as shape, color, texture, and spatial patterns [1,2,3]. In medical imaging, this process is particularly critical, as small visual patterns may indicate early-stage diseases. By utilizing machine learning and deep learning techniques, object recognition systems extract clinically relevant information from complex datasets and assist healthcare professionals by highlighting lesions, abnormalities, and other diagnostically significant structures. Object detection further extends this capability by not only identifying objects but also localizing them within images, supporting applications across X-rays, Magnetic Resonance Imaging (MRI), Computed Tomography (CT), ultrasound, and endoscopy images, as well as enabling patient monitoring tasks such as fall detection and posture analysis.

Despite its advantages, detection in medical diagnostics faces challenges including variations in object size, shape, and orientation, blurring effects, background noise, and the need for near-real-time processing with high reliability. Recent advances in deep learning and convolutional neural networks have significantly improved detection accuracy and robustness in complex clinical scenarios [4,5]. In real-world clinical practice, the timely and precise localization of pathological findings has a direct impact on diagnosis. Radiologists and clinicians often operate under time constraints, especially in emergency situations where stroke or trauma imaging can be life-preserving. Thus, computer-assisted detection systems cannot simply be seen as a technical aid but rather as a decision-support mechanism aimed at reducing delays in diagnosis, false detection errors, and variability in detection between observers. Among real-time detection frameworks, YOLO (You Only Look Once) has emerged as one of the most widely adopted approaches in medical imaging due to its efficiency and strong performance in precise object localization and classification.

YOLO was first introduced in 2016 as a single-stage detector, reimposing object detection as an issue of regression. Unlike two-stage detectors, such as R-CNN, YOLO performs localization and classification in one single pass of the neural network, thus achieving end-to-end detection with fewer computations [6]. The main benefit of using YOLO is achieved through its ability to process images at high speed without compromising diagnostic quality. However, since its inception, there have been continuous advancements in the YOLO family, ranging from YOLOv1 to YOLOv12, aimed at improving detection, robustness, ability to generalize, or efficiency. These advances have seen YOLO become increasingly applicable in medical diagnostic settings, especially in resource-constrained environments related to medical image analysis. While this section provides a high-level contextual overview of these developments, a detailed and systematically referenced examination of the architectural evolution of YOLO models, from v1 to v12 and beyond, is presented in Section 4.1, where version-specific innovations and their technical implications are discussed comprehensively.

Computer vision technologies are now central in contemporary healthcare and medical diagnostics today [7,8,9]. Diagnostic imaging, clinical monitoring, and image-guided surgical procedures are among the key areas where computer vision-based artificial intelligence systems enhance diagnostic precision and reduce human-related variability. Applications for the detection of tumors and their localization in radiological images [10,11,12], identification of lesions during endoscopic procedures [13,14], and monitoring of protective equipment compliance in the use within hospital environments are all examples of object detection applications and represent critical diagnostic and clinical support. Real-time detection frameworks such as YOLO represent a particularly good fit for these diagnostic tasks, where timely and accurate visual analysis is crucial for making clinical decisions, ensuring workflow efficiency, and maintaining patient safety.

Given the rising popularity of YOLO models and their implementation in the field of healthcare, there lies a definite requirement for a systematic synthesis of previous research work concerning their implementation, evaluation, and adaptation for use in medical diagnostics. As for the existing research work in this area, individual analyses have reported promising results. However, a systematic synthesis of existing work is required concerning a range of diagnostic applications in order to explore effective methodologies with a focus on existing limitations of their implementation. In this specific context, YOLO models are increasingly being utilized in the role of supportive medical tools to assist medical professionals for the purposes of efficient lesion detection, disease localization, and optimal workflow optimization with a range of existing imaging modalities. Motivated by the rapid evolution of YOLO architectures and the fragmented nature of existing healthcare-focused studies, this systematic review aims to provide a comprehensive and PRISMA-compliant synthesis of YOLO-based object detection models (v1–v12) in medical diagnostics, highlighting architectural trends, dominant application domains, recurring technical challenges, and evidence-based directions for clinically effective deployment, including the following:

- Analyze the yearly growth of research focusing on YOLO-based healthcare applications (Section 2.3).
- Categorize the reviewed studies according to their publication channels and article types (Section 2.4).
- Examine the evolution of YOLO architectures and discuss their relevance and impact on healthcare applications (Section 2.5).
- Provide a detailed overview of the evolution of YOLO and its major variants (Section 4.1).
- Investigate the popularity and adoption trends of different YOLO models reported in the literature (Section 4.2).
- Summarize the frequently reported challenges and the corresponding solution strategies proposed in primary studies (Section 4.5).
- Analyze the used image types in YOLO-based applications (Section 5.1).
- Analyze the detected disease types with YOLO-based applications (Section 5.2).
- Categorize and analyze the healthcare application domains where YOLO has been deployed, including medical imaging, disease detection, surgical assistance, and patient monitoring (Section 5).
- Review the performance metrics used to evaluate YOLO-based systems and analyze their frequency in the literature (Section 6).
- Identify current limitations, open challenges, and research gaps in applying YOLO models to healthcare problems (Section 7).
- Propose future research directions aimed at improving the real-world deployment of YOLO-based models in healthcare environments (Section 8).

This systematic literature review aims to offer an informative synopsis that will help and support researchers and developers as well as healthcare practitioners with the implementation of solutions that rely on the YOLO algorithm in the context of the development of new and innovative bionic–human-centered healthcare technologies that merge human interpretation with artificial perception. Unlike earlier narrative or domain-generic surveys, this research makes a unique contribution with its healthcare-focused analytical synthesis of various YOLO architectures (from v1 to v12), including their connections with important diagnostic constraints and application considerations. The manuscript’s unique blend of cross-version interpretation, methodological rigor evaluation, and performance analysis from 93 primary studies offers a framework for comprehending the impact of design decisions on model applicability in heterogeneous healthcare settings.

The rest of this paper is divided into the following sections. Section 2 details the research methodology applied to perform a systematic review. Section 3 provides background information on deep learning techniques in general and focuses on how CNNs form the basis for other object detection models. Section 4 explores a comprehensive review on YOLO techniques from its initial stages to recent developments. Section 5 offers a review on applying YOLO techniques within healthcare information systems. Section 6 provides light on metrics used to measure comparative results. Section 7 summarizes the most important challenges faced, according to the existing literature. Section 8 describes possible research directions related to bionic-oriented approaches in edge deployment, and explanation techniques. Finally, Section 9 concludes this document by highlighting findings from a systematic review.

## 2. Research Methodology

This section discusses the systematic procedure adopted in searching and selecting relevant literature on applying YOLO frameworks in healthcare environments. Although a formal protocol was not prospectively registered or published prior to conducting this review, the literature review is conducted following proper procedures as per guidelines for performing a systematic literature review. The review is performed in compliance with the Preferred Reporting Items for Systematic Reviews and Meta-Analyses (PRISMA 2020) principles [15] (in which the details are depicted in Supplementary Materials section). The used protocol includes specifying a research question, with the inclusion and exclusion criteria as depicted in Table 1, building a feasible search strategy, and synthesizing the retrieved result of studies. The research question in this review is stated as follows: “How have YOLO-based architectures been used in healthcare systems, and what have the consequences and weaknesses of the application in these systems been?” In order to consider relevance and quality in this review, the inclusion and exclusion criteria below are considered.

### 2.1. Search Strategy

In an effort to combine both quality and comprehensiveness, the collection of relevant literature for this review was performed through an exhaustive search across a host of reputable databases, including IEEE Xplore, PubMed, ScienceDirect, ACM Digital Library, SpringerLink, and MDPI. These were targeted because their coverage is focused on indexing peer-reviewed journals. Such sources have a higher level of scientific rigor and reliability than general search engines like Google Scholar, which include non-peer-reviewed or lower-quality sources. The search was limited to publications issued during the period from 2018 to 2026, reflecting the time period during which YOLO architectures from v1 to v12 have evolved and gained significant application in healthcare systems. With a view to maintaining the integrity and relevance of this dataset, all duplicate records identified across databases were systematically removed before the initial screening process. This was aimed at maximizing the retrieval of relevant studies while minimizing redundancy and possible biases in the sources selected.

**Figure 1:**
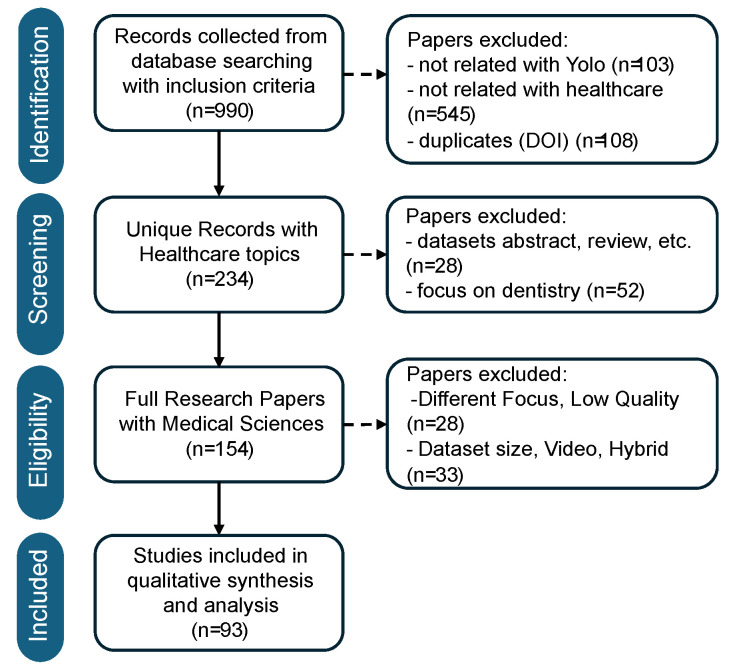
PRISMA flow diagram of literature review.

Figure 1 illustrates the systematic selection of studies adopted in this review, within a PRISMA-like workflow that ensures maximum transparency and reproducibility. A total of 990 records were retrieved from several academic databases using predefined inclusion criteria related to YOLO architectures and healthcare-related applications. Thereafter, 756 papers were excluded for irrelevant content related to YOLO itself (*n* = 103), failing to focus on healthcare applications (*n* = 545), and as duplicates detected by matching their DOI (*n* = 108). After such an initial filtering process, 234 unique records explicitly covering healthcare topics were forwarded to the screening stage.

In the screening and eligibility process, the remaining studies underwent more in-depth evaluation on the basis of the content. Dataset studies, abstract studies, review studies, or other forms of non-original contributions to the field of research (*n* = 28) or solely on applications to the field of dentistry (*n* = 52) were excluded to be consistent with the original review. Then, 154 full research articles in the medical sciences field remained. Another eligibility screening eliminated the studies having other research emphases or differing characteristics, such as inappropriate size for the studies conducted on the datasets, video-only studies, and combined models with architectures that were unrelated to YOLO architectures (*n* = 61 altogether). In the end, 93 studies met all the inclusion criteria and were used for the analysis consisting of the final collection to be used for the analysis of the development and medical applications of YOLO architectures ranging from v1 to v12.

### 2.2. Study Quality Assessment and Risk-of-Bias Evaluation

In order to advance the methodological rigor and the reliability of the synthesized evidence, a study quality and risk of bias assessment framework was used to examine the quality of the included articles. This framework assessed the studies along five domains, both in terms of technical robustness and applicability to healthcare. This selection of domains was intended to ensure a comprehensive investigation of the comprehensiveness, validation, reporting, and prevention of overfitting of YOLO-based studies in the healthcare domain.

1. Dataset Transparency involves the need for the source of the dataset, the adequacy of the sample size, and the class distribution to be reported. This is important for reproducibility and for comparing studies appropriately.
2. Validation Strategy, the splitting of the data into training and test sets should be transparent, and the use of cross-validation and external validation should be employed.
3. Performance should be reported using standardized metrics such as mAP, sensitivity, specificity, precision, and recall should be employed. In addition, confidence intervals should be used to validate the reliability of the performance of the model.
4. Assessment of overfitting involves the absence of data leakage, justification of data augmentation, and the use of external validation should be employed to validate the generalization of the model.
5. Clinical Relevance evaluates whether the model addresses real diagnostic needs, includes clinical interpretation of results, and considers practical deployment within healthcare workflows.

Based on these five domains, the methodological robustness of the included studies was systematically examined by using the scorings listed in Table 2. Given the heterogeneity observed across imaging modalities, dataset sizes, validation protocols, and reported metrics in the reviewed literature, applying this structured framework allowed us to comparatively evaluate study quality and identify recurring methodological strengths and limitations. This approach strengthens the rigor of the present systematic review and ensures that conclusions regarding YOLO architectures in healthcare diagnostics are grounded not only in reported performance values but also in methodological soundness and clinical applicability.

### 2.3. Publication per Year

**Figure 2:**
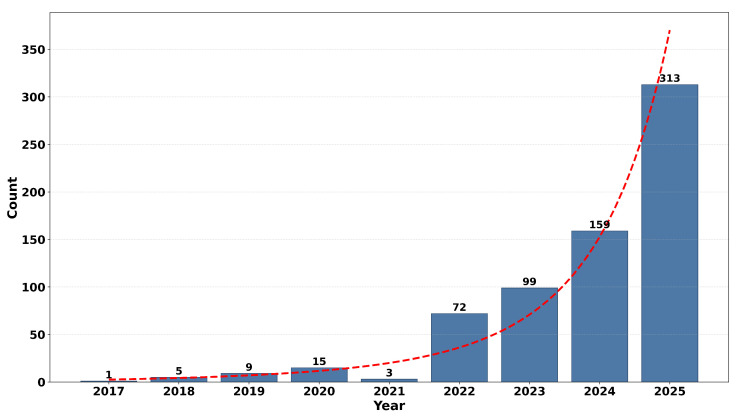
Distribution of publications per year in PubMed.

It can be noted from Figure 2 that the publication trend depicted in the graph reveals a significant and increasingly steep rise in publications related to YOLO-based architectures in healthcare systems [16]. During the period from 2018 to 2019, few publications were recorded, which reveals that the use of YOLO in the healthcare sector has merely started to take experimental shapes. From 2020 onwards, a gradual rise can be witnessed, which increased significantly in 2022 and further escalated in 2023 and 2024. The highest point in 2024 represents a rising interest in conducting more research work due to improvements in the architecture of YOLO, its efficiency, and growing applications in various areas of medicine, including image processing and disease diagnosis. Although a slight drop can be noticed in the graph related to 2025, this could be due to some partial figures that make less sense and not due to a decline in interest.

### 2.4. Yearly Trends of YOLO Publications

Figure 2 above shows the yearly distribution of PubMed-indexed publications on YOLO architectures in healthcare, and it indicates a strongly and rapidly increasing trend. During the years 2017 to 2020, there are few publications on this topic, showing that there is a preliminary exploration stage in relation to YOLO architectures in healthcare fields. There is a temporary slowdown in 2021, and this might be due to several reasons such as dataset constraints, validation in healthcare settings, and shifting towards more advanced architectures of the YOLO type. However, after 2022, there exists a remarkable and rapid escalation in healthcare publications, increasing from 72 in 2022 to 159 in 2024, with a peak of 313 in 2025. The dashed trendline in this figure has been used to emphasize the exponential escalation in YOLO architecture-related healthcare publications and illustrates a strongly emerging trend in YOLO architectures being employed in healthcare areas such as medical image processing, disease diagnostics, and healthcare support systems in rapidly increasing levels in recent years.

It should be noted that while conducting the yearly publication trend analysis as depicted in Figure 2, only publications indexed by PubMed are considered. Although it is true that PubMed has excellent coverage of biomedical research, it is also true that limiting our analysis to this particular source may cause selection bias because of insufficient coverage of publications focused on engineering. Thus, it is to be noted that the publication growth trends depicted above are mainly focused on healthcare-related research.

### 2.5. Article Groups

**Figure 3:**
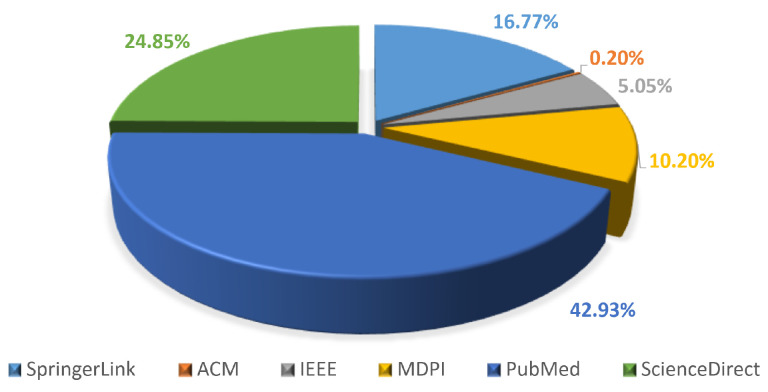
Publication channels of the related literature.

Figure 3 describes the distribution of academic publications on YOLO architecture versions 1–12 in healthcare systems across the top academic publication channels from 2017 to 1 January 2026. As observed from this, the maximum number of papers are indexed at PubMed (42.92%), indicating widespread use of YOLO architecture in the biomedical and healthcare research community. This is closely followed by ScienceDirect (24.85%) and SpringerLink (16.76%), indicating significant contributions from engineering and computer science-related publications, which often feature multidisciplinary healthcare and artificial intelligence studies. The MDPI journal group ranks next, with 10.20% contributions, emphasizing its importance in publishing open-access content in the areas of medical imaging and deep learning. IEEE (5.05%) and ACM (0.20%) are smaller, suggesting that YOLO architecture in healthcare applications is often indexed in medical and multidisciplinary databases but less often in computer science and software libraries. From this figure, it is clear that most papers on YOLO in healthcare applications are indexed in databases with significant biomedical and applied artificial intelligence content, thereby underpinning the importance of an academic and wide-scale search in systematic reviews.

The main search query is: (“YOLO” OR “You Only Look Once”) AND (“healthcare” OR “medical” OR “medicine” OR “health” OR “clinical” OR “patient monitoring” OR “disease detection”). These queries were tested on the Title, Abstract, and Keywords fields. After testing the queries on the appropriately selected databases, 990 publication matches were obtained. The searching expressions were tailored to suit each of the searching capabilities offered by each database. Filtering options used included language (English) and publication type, which is a journal.

### 2.6. Previous Reviews and Distinctive Properties

There have been several review studies in the past that have analyzed, respectively, the available YOLO-based techniques for the detection of objects in a wide variety of fields of application, ranging from autonomous vehicles to agriculture, industry inspection, UAV technology, and computer vision applications in general. As indicated in Table 3 above, the previous literature reviews have largely employed methodologies that were either non-systematic or partly systematic in nature, with the aim of covering either the topic of object detection or the application of the technology of deep learning in general, with little emphasis placed directly upon healthcare applications in particular.

By contrast, only few previous studies clearly position their application domain within healthcare, and these tend to be either narrow to specific medical imaging tasks (e.g., oncology or radiology) or adhere to an older generation of YOLO models (e.g., YOLOv1–YOLOv8). In addition, several state-of-the-art reviews use a limited portfolio of scientific databases. Many rely on a single indexing service such as PubMed or Scopus alone, which again limits the coverage and introduces a selection bias. Finally, many earlier surveys exclude the very latest YOLO variants or fail to comprehensively represent the rapid architectural advances of the latest YOLOv9–YOLOv12; therefore, they also have a reduced relevance to contemporary research trends.

The current study is differentiated by four defining properties. First, this review assumes a health-centric perspective by undertaking a systematic review of YOLO architectures only for applications inside health systems, such as medical imaging, disease detection, diagnostic support, and clinical decision-making. Second, this review covers a wider and more comprehensive academic search by combining multiple major scholarly databases and digital libraries, including but not limited to IEEE Xplore, Scopus, PubMed, SpringerLink, and ScienceDirect. That means a wide coverage inter- and intra-disciplinary high-impact studies has been deemed indispensable. Third, the most recent literature is reviewed by explicitly focusing on YOLO architectures from v1 to v12, therefore capturing state-of-the-art methodological developments and new health applications. Finally, unlike many non-systematic or narrative reviews that are common in the past, this paper is underpinned by an SLR methodology, guaranteeing transparent selection of studies, reproduction, and an objective synthesis of findings.

Combined, these singular characteristics place the proposed review in a unique position as comprehensive, current, and methodologically sound SLR that will fill a critical gap in the literature by providing a single and healthcare-focused review of YOLO architectures across their full evolutionary spectrum. This study distinguishes itself by providing a comprehensive and PRISMA-compliant systematic review covering the full evolution of YOLO architectures from v1 to v12, including recent attention-based and efficiency-oriented variants that are often excluded in earlier healthcare-focused reviews. Based on 93 rigorously screened studies, it extends beyond narrative description by offering quantitative analyses such as model adoption trends, bibliometric mapping, modality–disease categorization, and structured challenge–solution synthesis. Moreover, it incorporates a clinical deployment perspective, addressing dataset limitations, computational constraints, and generalization challenges relevant to real-world healthcare integration.

## 3. Deep Learning Approaches and Convolutional Neural Networks

One of the rapidly developing areas of Artificial Intelligence is the advent of Deep Learning, which has been facilitated by tremendous growth in computing capability, available dataset, as well as the evolution of new architectures in neural networking technology. Amongst various neural net models, Convolutional Neural Networks or CNNs have been the workhorse in dealing with image analysis tasks of various kinds in the field of Artificial Intelligence and Machine Learning. They are highly useful in dealing with grid-based data, as in the case of image analysis, as they learn hierarchical features from raw pixel values in a unique manner.

**Figure 4:**
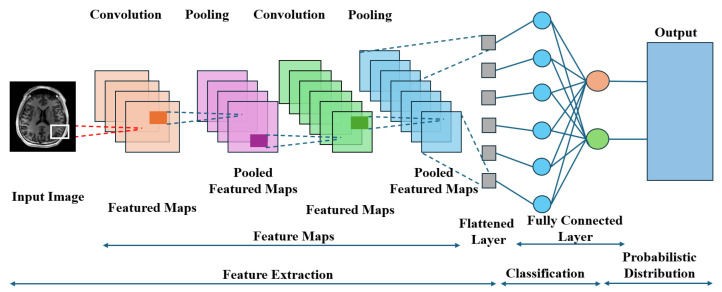
Convolutional neural networks.

Figure 4 shows that CNNs are designed to mimic the human visual system, whereby several layers of interconnected neurons collaborate to extract meaningful patterns from visual input. Typically, a CNN is composed of convolutional layers, pooling layers, and fully connected layers. In convolutional layers, filters are applied to the input data in order to obtain feature maps, which capture local patterns such as edges or textures. The pooling layers reduce the spatial dimensions of the feature maps, hence making the computation efficient and allowing the network to become invariant to factors such as position or scale. Finally, these high-level features from the convolutional and pooling layers are interpreted by fully connected layers in order to perform a classification or regression task, depending on the application.

In the medical industry, CNNs have demonstrated outstanding capabilities in several medical applications. Currently, in medical imaging, CNNs are employed for cancer detection and classification in medical images such as CT scans, MRIs, and X-rays. They also support organ or lesion segmentation, which helps improve medical diagnosis and treatment plans. Apart from medical imaging tasks, CNNs are employed in pathology to examine tissue samples, in dermatalogy to diagnose skin disorders, and in ophthalmology to evaluate patients with retinal disorders. Furthermore, CNNs also contribute in predictive analytics for analyzing patterns in electronic medical records and even in behavior analysis in patient monitoring systems in hospitals in real time.

The design principles of CNNs highlight the significance of the structured input and the output interpretation. The inputs to a CNN are typically images or more general multidimensional arrays, where the spatial layout of the data is maintained to enable efficient convolutional computations. The architecture has been designed to leverage spatial correlations within the data through the use of smaller kernel filters scanning the input image. Output of a CNN task also typically depends upon what task a CNN has been trained upon. In classification tasks, the output is a probability distribution over predicted classes, while detection, segmentation tasks yield output bounding boxes, masks, or heat maps representing the location and extent of the object of interest.

A further important strength in CNNs is their capacity to learn from large-scale annotations in a dataset through backpropagation and gradient descent. This is different from traditional approaches that involve feature engineering. Here, CNNs learn important features automatically as opposed to traditional approaches that were manual. This makes CNNs flexible in this regard as well. Additionally, their performance is largely dependent on large-scale annotations and careful selection of other hyperparameters such as learning rate, kernel size, and depth. Furthermore, other sophisticated approaches such as batch normalization, dropout, and augmentation in this regard can be utilized in CNNs to enhance flexibility and efficacy in such settings as a modern health system.

## 4. Overview of YOLO Architectures

Extending the vital usage of Convolutional Neural Networks for the processing of visual data, models for the detection of objects have been developed that further increase the usage of CNNs for the detection of more than one object in an image. These models enable the processing of complex visual data in real time. Among these models, there is one type of model that has recently gained immense popularity owing to the substantial balance that this model provides between speed and accuracy, making this model highly suitable for medical applications.

It was a very big improvement; this provided a real-time solution for object detection. YOLO introduced at the IEEE Conference on Computer Vision and Pattern Recognition, 2016 by Joseph Redmon et al. [25] was significant in that it looks at an image only once. This is a drastic change from the methods that have used a sliding window approach. This means that a classifier runs many times within an image. Other approaches had a two-stage method where a model predicts regions and then a classifier is applied on these regions.

Although the following figures primarily illustrate architectural evolution, they are included to provide historical and technical context necessary for understanding the progression of YOLO systems. Since this review spans versions from YOLOv1 to YOLOv12, visualizing structural changes helps clarify how improvements in detection mechanisms, efficiency, and feature representation have influenced healthcare applications. These figures therefore support interpretability of version-specific performance trends rather than serving as purely engineering illustrations.

**Figure 5:**
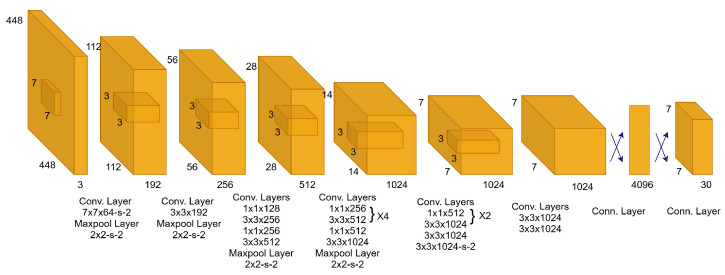
YOLO architecture.

The architecture of YOLO in Figure 5 follows a single-stage, end-to-end design that processes the whole image within a single pipeline. Early convolutional layers extract low-level visual patterns, including edges and textures, while the pooling reduces spatial complexity and enhances efficiency. As deeper layers are stacked on top of them, early features become transformed into rich semantic representations, allowing the model to represent object-level structures and contextual relationships in the whole image. In the later stages, such feature maps are generated as small in spatial extent but semantically powerful and are mapped directly to the detection output. Instead of region proposal generation, in a single stage, it predicts the object presence, object location, and class information. As a result, each prediction is conditioned on the entire image context, leading to fast and reliable object detection. This is what sets YOLO apart from other models in the real-time analysis of healthcare imaging, surveillance, and autonomous systems.

“YOLO” refers to the acronym for the real-time object detection algorithm family whose evolution has been remarkable since the time of its launch. Learning from the basic concepts of deep learning models and the architecture of the Convolutional Neural Network (CNN) model, the “YOLO” algorithm was built while keeping in mind the need for efficiency. “YOLO” models have found applications in several fields that demand fast processing for accurate detection, such as the medical field. This section introduces the reader to the “YOLO” algorithm in detail.

**Figure 6:**
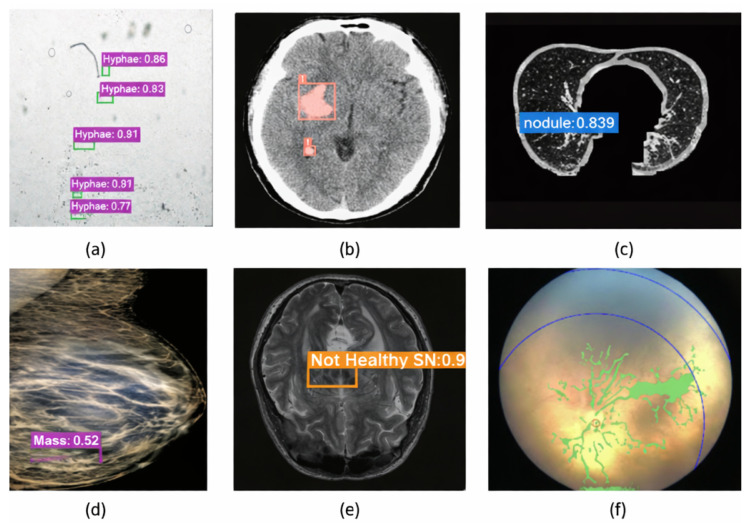
YOLO in different medical images applications: (**a**) superficial fungal structure detection in microscopic images, (**b**) intracranial hemorrhage volume localization in brain CT, (**c**) lung nodule detection in thoracic CT, (**d**) breast mass detection in mammography, (**e**) Parkinson’s disease-related structural abnormality detection in MRI/DaTscan, (**f**) vascular feature extraction for retinopathy of prematurity (ROP) screening.

Figure 6 presents the application of YOLO-based medical imaging approaches used in the studies reviewed across diverse clinical modalities. The detection of fine-grained morphological patterns of fungal structures in microscopic images (a) using regional convolutional networks demonstrates the high sensitivity of these methods even at the cellular level [26]. The precise localization of hemorrhagic regions in brain CT scans (b) through YOLOv8-based rapid detection mechanisms highlights the algorithm’s potential for integration into time-critical emergency diagnostic workflows [27]. Automatic identification of small, low-contrast nodules in thoracic CT images (c) via multi-scale feature fusion techniques is essential for the early diagnosis of lung pathologies [28]. In breast imaging (d), the detection of lesions captured through some modalities using YOLO–ViT hybrid systems underscores the advantages of combining deep feature extraction with global context modeling [29]. The detection and segmentation of structural abnormalities associated with Parkinson’s disease in brain MRI/DaTscan data (e) further demonstrate the expanding role of deep learning in the early recognition of neurodegenerative conditions [30]. Finally, the automated extraction of vascular network structures from retinal images (f) highlights the potential of these methods to enhance clinical decision-support accuracy in screening for retinopathy of prematurity [31]. Collectively, these examples illustrate the adaptability of deep learning models to diverse anatomical structures, imaging modalities, and clinical scenarios.

### 4.1. Evolution of YOLO (v1 to v12 and Beyond)

**Figure 7:**
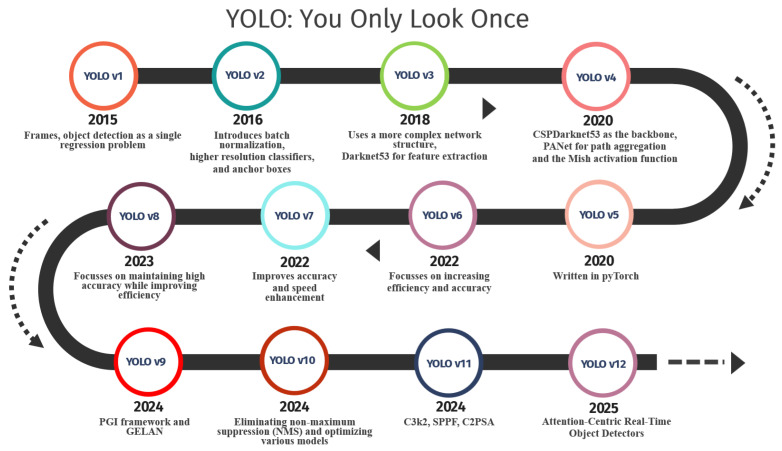
Evolution timeline of YOLO architectures (v1–v12) and their progressive Impact on clinical object detection performance.

Figure 7 represents YOLO’s timeline, which marks a continuous enhancement process starting from speed optimization and moving toward precision, optimization, and complex model designing. YOLOv1 introduced the concept of handling object detection with a single regression task, which made real-time object detection possible. Later, YOLOv2 and YOLOv3 improved localization and feature representation by adding components in their model architecture. On the other hand, YOLOv4 introduced the concept of refined backbone networks, feature aggregation, and more efficient activation Gaussians that resulted in substantial improvement. After YOLOv5, object detection algorithms moved toward making these models more user-friendly, efficient, and easily scalable by the adoption of newer deep learning frameworks. From YOLOv6 to YOLOv8, the precision-speed balances were improved along with a focus on efficient deployment and usage. Finally, models from YOLOv9 to YOLOv12 came up with new ideas regarding learning and designing through training, along with a focus on attention mechanisms. The timeline for YOLO shows the transition from speed-sensitive object detectors to a full-fledged efficient as well as real-time object detection mechanism that is capable of handling complex tasks such as healthcare and autonomous driving.

Understanding the evolution from YOLOv1 to YOLOv12 is particularly important in healthcare diagnostics, where architectural refinements directly influence clinical applicability. Early versions primarily prioritized real-time detection speed, whereas later generations progressively incorporated multi-scale feature fusion, anchor-free mechanisms, decoupled detection heads, attention modules, and efficiency-oriented backbone designs. These developments are closely related to healthcare-specific challenges such as small lesion detection, imbalanced datasets, domain generalization, and deployment under limited computational resources. Therefore, analyzing YOLO’s architectural progression provides not only a historical overview but also a clinically relevant framework for selecting appropriate variants based on diagnostic complexity, dataset scale, and operational constraints.

- Since its launch in 2015 by Joseph Redmon and his colleagues, the **YOLOv1** approach has undergone various versions one after the other, which transformed the way objects are detected with the introduction of real-time end-to-end processing, which was highly unconventional compared to the then existing multi-stage object detection methods. Contrary to the then existing approaches like R-CNN or its successors, which emphasized the need to predict the region proposals followed by the calculation of the class probabilities, YOLOv1 tackles the entire problem of detecting objects as a unified regression problem in which the network predicts the bounding boxes and class probabilities for the entire image at once, hence allowing real-time processing at faster speeds with improvements in efficiency. For the network to work on the entire input image, it splits the image into an S × S grid, which identifies the bounding boxes and computes the corresponding class probabilities for each cell that contains the center points of the objects to be identified. Since the approach tackles the entire problem statement as a regression problem rather than focusing on the various region proposals for identification, it allows the network to overcome the various computation hurdles faced in the region proposal-based methods with higher accuracy.
- **YOLOv2 (YOLO9000)** was proposed by Joseph Redmon et al. as a major improvement to the original YOLO framework. Though the original YOLO network shows the capability to perform real-time object detection at incredible speed, it faces drawbacks regarding localization accuracy and lower recall with respect to two-stage detectors such as Faster R-CNN. YOLOv2 removes these drawbacks with the use of various architectural enhancements and learning techniques to improve the detection accuracy as well as speed. One of the biggest improvements brought about by YOLOv2 is the application of batch normalization on all the convolutional layers that improve the mean average precision by above 2% with greater stability in the training procedure. Furthermore, the network substitutes the use of fully connected layers with anchor boxes similar to the Faster R-CNN model, enabling it to predict the bounding boxes more freely. This enables it to perform better on objects with varying dimensions and ratios.
  Some initial healthcare applications utilized YOLOv2 for addressing the task of cell detection in microscopy and radiographic imaging. The SO-YOLO model utilized the YOLOv2 architecture for cell detection within white blood cells using Fourier Ptyographic Imaging, in which high-resolution cell positioning was accomplished for microscopic imaging with high accuracy [32]. The YOLOv2 architecture was further applied for the automatic detection of Alzheimer disease, presenting its efficacy in healthcare applications in MATLAB’s image label tool [33]. Furthermore, the application of YOLOv2 was incorporated in an end-to-end deep learning network for detecting microbleeds within the cerebrum, in which YOLOv2 was proficient in maintaining an optimal balance between sensitivity and specificity for healthcare imaging analyses [34].
- **YOLOv3** represents a large leap in the series from its predecessors, giving a significant boost to detection accuracy and multi-scale object recognition capability. One of the key novelties of YOLOv3 is its new backbone network, Darknet-53, consisting of 53 convolutional layers with residual connections. While allowing for deeper feature extraction, this gives a good trade-off between speed and accuracy. Besides that, YOLOv3 uses FPNs to make predictions at three different scales, which helps it detect small, medium, and large objects more reliably than earlier YOLO versions, which suffers from multi-scale object detection. Unlike the previous versions that rely on softmax classifiers, YOLOv3 uses independent logistic classifiers for multilabel prediction. This significantly enhances the capability of the model for dealing with overlapping classes and increases its viability in real-world complex domains like healthcare imaging. YOLOv3 further enhances the anchor box approach to object detection, which was proposed with YOLOv2, with the use of fixed bounding box priors and making detections with respect to the anchor boxes, thus making detections more precise. All these enhancements have contributed to the effectiveness of the YOLOv3 model in applications dealing with complex medical images that often require the accuracy of detecting various types of abnormalities at various scales. It should be noted that the efficiency and accuracy of YOLOv3 make it the basic model for various applications of medical diagnostics in real-time analysis.
  YOLOv3-based studies demonstrate its adoption in complex radiological analysis tasks rather early. Dual-view mammogram matching with YOLOv3 embedded enhances breast mass detection by leveraging complementary information from multiple imaging perspectives [35]. Further, studies on automated vertebral fracture detection on lateral spine radiographs indicate that the YOLOv3-based approaches are sensitive to generalizability of ground truth, emphasizing the impact of annotation quality and dataset variability on detection performance [36].
- **YOLOv4** is a major improvement in the YOLO series and includes many architectures and training design changes to improve speed and accuracy simultaneously. A major distinctive feature of YOLOv4 lies in its use of two techniques called “Bag of Freebies” and “Bag of Specials.” “Bag of Freebies” involves several techniques to improve training without slowing down processing speed during inference, such as data augmentation techniques like Mosaic and CutMix image segmentation techniques. The Mosaic image technique involves merging four training images into a single image to enable better detection of smaller objects and understanding of inter-object relationships. In contrast, CutMix involves cutting parts of different images and blending them together to make different versions of one image to improve robustness to different versions of objects in different images. On the other hand, “Bag of Specials” involves several techniques like activation function ”Mish” and ”Spatial Pyramid Pooling,” which improve inference speed and precision, respectively. ”Mish” activation helps improve generalizability of objects in different pictures, while Spatial Pyramid Pooling improves the receptive field of the network to perform better on objects of different sizes.
  Initial medical image processing and analysis studies based on YOLOv4 have proven its efficiency in microscopic and radiological image analysis. The automatic detection of superficial fungal infections based on YOLOv4-regional convolutional neural networks has demonstrated correct infection pattern localization in microscopic images for infected patients and healthy individuals accurately [26]. Furthermore, for breast lesion detection and classification in images, the use of a YOLO-based Computer-Aided Diagnosis system with Vision Transformer models has increased efficiency, showcasing its feasibility to be modified according to state-of-the-art feature extractors such as Vision Transformer models [29]. Moreover, designing an intelligent system for malaria pathogen detection has utilized YOLOv4 and proven its efficiency in identifying parasites accurately in microscopic images [37].
- **YOLOv5** is the first major version implemented natively in PyTorch and need PyTorch >=1.7 installed to the computer. This shift makes it significantly more accessible for developers and researchers to train, fine-tune, and deploy. But this has several advantages such as ease of experimentation, easier development, and better alignment with the general Python >=3.8 data science environment. Moreover, the YOLOv5 models have the differentiability in the scale of the models or the size of the models, meaning that the size of the models can range from Nano to Extra Large based on the requirements of the users who wish to use it, from the size of the devices it can fit into to the size of the computers with high processing capabilities it can handle optimally. In addition, the Ultralytics developed the YOLOv5 in an appropriate repository with good and adequate documentation, and it already included the weights in the models for training and validation purposes, making it more accessible and acceptable in its use in different applications in the shortest time possible. Moreover, the YOLOv5 includes several different improvements in its architectures such as high-quality data augmentation, computation of the anchor boxes, and appropriate hyperparameters.
  Some studies have comprehensively examined YOLOv5 for the analysis of medical images. The contribution of MedYOLO is presented as a YOLOv5-based framework for medical object detection. Highlighted is the improvement brought about regarding localization capabilities on clinical images [38]. Among similar lines, YOLOv5 has been employed for the analysis of breast lesions. The ability of YOLOv5 for proper detection and classification has been confirmed for suspicious areas determined from mammograms [39]. In addition, YOLOv5 improvement methods were raised for the identification of irregular pelvic radiographs, suggesting that all sorts of complementary information inputs could enhance localization capabilities during medical imaging analysis [40].
- **YOLOv6 and YOLOv7**: YOLOv6 is characterized by its emphasis on industry-level applications and has a structure that is optimized to perform well both from a performance and efficiency standpoint. It has a decoupled head, which is a design philosophy that breaks up the processes of object classification and localization to reach a better accuracy level, mainly for smaller-sized objects. Another new feature in the YOLOv6 model is a re-parameterized backbone network, which improves the model’s representational capabilities during training and is also optimized for faster inference capabilities. Therefore, it can easily serve to meet the demands of precise and fast processing required during real-world applications and experiments. Conversely, the main highlighting feature of YOLOv7 is a list of architectural novelties and sophisticated learning approaches that help to define a model that is at the edge of the performance chart for the task of object detection. One of its most striking features is the use of the concept of “extendable efficient layer aggregation networks,” which improves the model’s learnability along with computational efficiency. Another feature highlighted by YOLOv7 is that it uses a compound scaling approach that enables the scaling of model depth and width while keeping the optimal model structure intact.
  YOLOv6-related studies also emphasize the progression in the accuracy of detection in the thoracic and neuroimaging fields. The LUNG-YOLO method improves YOLOv6 with multiscale feature fusion, attentional fusion, and cross-layer aggregation, and offers improved lung nodule detection capability in chest CT imaging [41]. In addition, YOLO model benchmarking on intracranial hemorrhage detection with diverse CT imaging sources also proves the consistent robustness and reliability of YOLOv6-related implementations [42].
  Some studies have also investigated the feasibility of employing the detection frameworks provided by the YOLOv7 model in musculoskeletal and dermatological tasks. The development of AI algorithms with the capability of detecting pediatric wrist fractures indicates the potential of the YOLOv7 model in terms of able detection of the freely available radiographic databases, thus pertinent to clinical practice [43]. The other one, the detection of the lesion through the use of the YOLO-MR meta-learning framework, utilizes the capabilities of the YOLOv7 model to deal with the balance between images, thus more effective, especially when dealing with the attainability of the images [44]. In contrast to this, the studies regarding the classification of skin cancer employing the aspects of the traditional machine learning model serve as the perspective [45].
- **YOLOv8** is a greatly improved version of the YOLO model yielding a radical transformation in architectural design from that of its previous counterparts. Gone are the days when models like its predecessors used to employ an incremental model design on the Darknet-based structure. There is a radical change in the newly introduced model regarding classification and localization, which is now convolutional and anchor-free, featuring a decoupled head. A further important aspect of this model becoming anchor-free is that it makes the detection process much simpler compared to previous models, which required a greater deal of care concerning the definition of anchor box configurations. Another important feature is its flexibility and modularity, which is greatly beneficial for healthcare applications involving the detection of lesions that could be of different sizes and sometimes irregular in pattern.
  Recent works further verify the superiority of deep learning-based object detection models for various radiological tasks. A smart system for the evaluation and classification of rib fractures further proves the accuracy and potential of deep learning models in bone fracture location and corresponding evaluation from radiographic images [46]. Similarly, the application of YOLO-v8 in detecting micro-afflications in mammographic images yields improved performance for significant clinical implications for detecting breast cancer in its initial stages [47]. Furthermore, optimizing feature interaction has proved the significance of its impact in joint lesion detection, further stating its importance in proper feature modeling for optimal pathological feature recognition in Musculoskeletal Imaging [48].
- **YOLOv9**, launched in early 2024, also marks a major upgrade from its predecessors, as it combines state-of-the-art architectural advancements to further improve both accuracy and efficiency. One thing that stands out about YOLOv9, however, is that it also incorporates a new mechanism called Generalized Efficient Layer Aggregation Network (GELAN), which enhances features from various network layers. Indeed, unlike its predecessor, which utilizes a conventional backbone network, GELAN allows YOLOv9 to strike a balance between efficiency and accuracy through better multi-scale features. Its improved feature aggregation is crucial for capturing multi-scale information, which is vital for detecting medical abnormalities of varying sizes (e.g., small microcalcifications and large tumors). Additionally, YOLOv9 employs a new dynamic label assignment strategy, designed to improve the training process by making object detection supervision more consistent and robust, particularly in dense and complex scenes often encountered in healthcare imagery.
  A study on benchmarking YOLO models for intracranial hemorrhage detection focuses on testing the ability of the new YOLO variants such as YOLOv9 concerning their robustness and effectiveness in detecting intracranial hemorrhages through CT scans [42].
- **YOLOv10** brings a new paradigm to real-time object detection with a focus on end-to-end efficiency. A key distinction is its use of “consistent dual assignments” during training, which allows the model to operate without non-maximum suppression (NMS) during inference. This NMS-free approach significantly reduces inference latency, making YOLOv10 particularly suitable for applications where speed is critical. This is particularly beneficial for real-time applications like surgical instrument tracking or fall detection. YOLOv10 also has a holistic model design approach based on efficiency and accuracy, which focuses on reducing the latency of a lightweight classification head and spatial-channel decoupled downsampling.
  Current literature proves the efficiency of using YOLO-based models for critical medical imaging. Specifically, the benchmarking of YOLO models for the detection of intracranial hemorrhage in heterogeneous CT images emphasizes the real-time capabilities of such models for time-critical medical applications [42]. At the same time, the application of deep learning models for the detection of pancreatic cystic neoplasms using the Mamba architecture in medical images exemplifies the usage of efficient models to address the complex spatial relationships within the images [49], providing complementary information on the applicability of medical image detection models within healthcare frameworks.
- **YOLOv11** represents one of the most recent evolutions in the family of object detection models called YOLO. It brings about conspicuous improvements related to both performance optimization and real-world deployment, especially in resource-constrained systems typical of healthcare. Based on the architectural refinements of its predecessors, YOLOv11 insists on modularity, simplicity, and efficiency. A more flexible backbone is adapted, together with a scalable detection head. Dynamic neural network architectures that adapt the computation paths depending on the input complexity are used, enabling faster inference on simpler cases while maintaining high accuracy for more challenging ones. This dynamic behavior provides benefits in healthcare imaging, where diagnostic images may vary greatly in their native complexity or resolution.
  Recent improvements that correspond to YOLOv11 indicate a shift towards multi-objective and clinically optimized detection frameworks. A multi-objective deep learning approach for lung cancer detection enhances tumor classification, precise localization, and overall diagnostic efficiency in computed tomographic images, reflecting the capability of next-generation YOLO architectures to jointly optimize multiple clinical tasks within one unified pipeline [50].
- **YOLOv12** is an emerging object detection model, which expands the YOLO series by introducing an attention-centric architecture to keep, as well as improve, the real-time processing capabilities handled by the earlier CNN-based YOLO models [51]. First introduced in the “YOLOv12: Attention-Centric Real-Time Object Detectors” work, it supports not only object detection but also classification, segmentation, and other vision tasks, and can be deployed across diverse platforms including NVIDIA GPUs and edge devices using frameworks like Roboflow Inference. This allows for better contextual modeling, improving the model’s ability to differentiate between subtle lesion features and complex background tissue. The processing speed at which YOLOv12 conducts its inferences is on par with its predecessors while being faster, due to its attention-centric architecture, which helps in better contextual representation than its predecessor models.

### 4.2. Analysis of YOLO Version Usage in Healthcare Applications

It should be noted that despite the rapid evolution of the YOLO family of object detection algorithms from the first version to the latest, the majority of the versions have been actively used in healthcare research. Some of the older versions have been the first choice in the study of clinical imaging due to their proven reproducibility and benchmarking history, while the latest versions have been the subject of emerging object detection algorithms in the field of healthcare. Therefore, in the following section, the evolution of the YOLO family of object detection algorithms and their level of evidence, both experimentally validated and using real-world data, are discussed.

**Figure 8:**
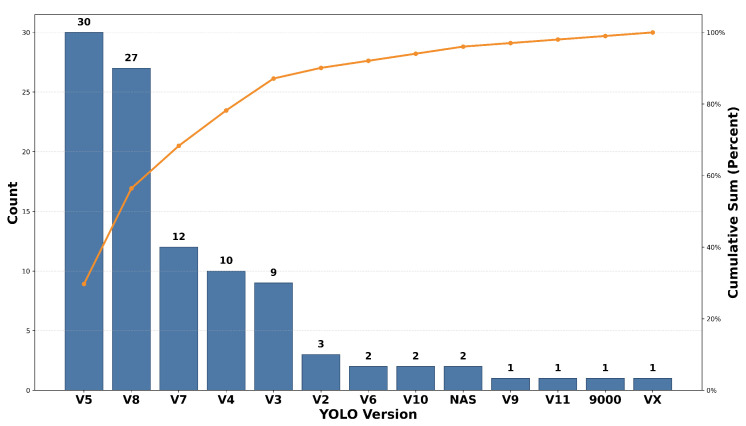
Frequency of YOLO versions used in healthcare studies, reflecting model adoption trends in clinical diagnostic applications.

Figure 8 indicates the use or adoption frequency of the preferred architectures of YOLO models and their cumulative sum as shown in orange line among the studied literature, which shows that YOLOv5 and YOLOv8 are the mostly used models in the literature. The dominant use of YOLOv5 indicates that the newer version of the model has quickly gained popularity among researchers because of its enhanced performance and usability. Although the newer version of the model has gained popularity, the dominant use of YOLOv8 indicates that the version has maintained its important position among the models because of the support it has maintained and the ease with which it can be used. Another important perspective here indicates that the use of the older versions of YOLOv1, YOLOv3, and YOLOv4 also has some significant value because the models are preferred for sustainability or reproducibility reasons in some applications. On the other side, the widespread adoption of YOLOv5 and YOLOv8 in clinical studies reflects a practical trade-off between detection accuracy and computational complexity. While larger or more recent variants may provide marginal accuracy gains, they often incur higher memory usage and inference latency.

The cumulative curve further shows that these five variants alone account for almost 90% of all the included studies, revealing a strong inclination of research toward mature, well-supported, and performance-optimized variants of YOLO. The earlier variants, such as YOLOv2, on the other hand, are rarely used in healthcare applications, let alone other less commonly adopted or experimental YOLO variants like YOLOv6, YOLOv9–YOLOv11, NAS-based YOLO, and custom variants, including 9000 and VX. This distribution indicates that healthcare applications tend to favor stable and widely validated YOLO architectures, likely due to their reliability, pre-trained models, and ease of integration into medical imaging pipelines.

### 4.3. YOLO Evolution in Healthcare Applications

One of the main analytical contributions of the present work is the examination of the architectural evolution of the YOLO series, from v1 to v12, through a clinically informed perspective, continuously advancing the state of the art in the field of real-time object detection in the medical and healthcare domains in terms of speed, diagnostic accuracy, and computational efficiency. In the medical and clinical field, where the support of clinical decisions is crucial, new versions of the YOLO series have introduced sophisticated architectural mechanisms to address the limitations of the earlier versions, especially in the context of the detection of small lesions, overlapping anatomical structures, and partially occluded pathological areas in medical imaging modalities such as X-ray, CT, MRI, ultrasound, etc.

- **Early Versions (YOLOv1–YOLOv3)** introduced real-time detection and multi-scale feature extraction. This helped in the detection of large abnormalities such as lung consolidations and visible skin lesions. Later updates helped in better performance in the detection of small objects such as pulmonary nodules and microcalcifications.
- **Intermediate Versions (YOLOv4–YOLOv7)** focused on the optimization of the speed-accuracy tradeoff by employing the CSPNet and augmentation techniques, enhancing the robustness of the models for limited medical datasets. These models were used for a variety of medical computer vision tasks, such as breast cancer, COVID-19, fracture, and polyp detection, performed during endoscopy, in addition to the deployment of these models in hardware-efficient clinical context.
- **Modern Versions (YOLOv8–YOLOv12)** introduced anchor-free detection, attention mechanisms, programmable gradient strategies, and NMS-free inference. These advancements reduce false positives, preserve deep feature information, and enable faster inference—critical for real-time clinical tasks such as intraoperative guidance, emergency imaging analysis, and dense lesion detection in histopathology.

### 4.4. Performance Comparisons with Examples in Healthcare

Recent structural enhancements translate into measurable performance gains in complex medical imaging datasets. For example, in a Lung Cancer Detection study, YOLOv11 demonstrated superior capability in detecting subtle lung tumors, achieving an mAP of 96.26% and an IoU of 95.76%. This outperformed YOLOv10 (mAP: 95.23%, IoU: 94.28%) and YOLOv9 (mAP: 95.70%, IoU: 94.10%) [50]. The improvement was attributed to transformer-based attention layers and deeper feature pyramids. Similarly, in a Blood Cell Detection study, YOLOv11 achieved the highest mAP50 of 0.958, followed by YOLOv10 (0.944). Both surpassed YOLOv9 (0.933), YOLOv8 (0.930), and YOLO-NAS (0.904) [17].

Although YOLOv11 and YOLOv10 dominate complex benchmarks, earlier versions remain competitive in specific medical use cases [42]. For example, in intracranial hemorrhage detection from CT scans, YOLOv8 achieved the highest overall mAP (0.96) and recall (0.92) across hemorrhage classes. While YOLOv10 improved computational efficiency by eliminating NMS, YOLOv8 delivered more robust diagnostic accuracy for that task. Similarly, in relatively simple tasks such as basic facemask detection without occlusion, older architectures like YOLOv7 (mAP50: 0.927) remain highly effective due to their lighter structure and sufficient feature extraction capacity.

### 4.5. Identified Challenges in Previous Studies

Based on the analysis of the 93 publication papers in this research, the essential challenges in medical image processing and computer-assisted medical diagnosis are revealed in Table 4 below in which multiple challenges may co-occur within a single study; therefore, percentages are not expected to sum to 100%. Based on the research results, it can be concluded that the current research faces some fundamental challenges, such as the use of limited or imbalanced data, variance or noisy images, the complexity of multimodal data fusion, the issue of domaintransfer, the issue of fine-grained lesion localization, and high computational complexity.

Nevertheless, the literature presents a broad spectrum of solution strategies, varying from sample-efficient methods such as transfer learning and data augmentation to state-of-the-art representation models, including CNN–Transformer hybrids and multimodal fusion mechanisms; and from YOLO-based rapid detection techniques to U-Net-style segmentation architectures and lightweight model designs with the intention to speed up computation. Table 4 maps these challenges to their methodological counterparts in an overall manner, thus providing an understandable and structured synthesis of prevailing trends, research gaps, and strategic directions for future solutions.

**Figure 9:**
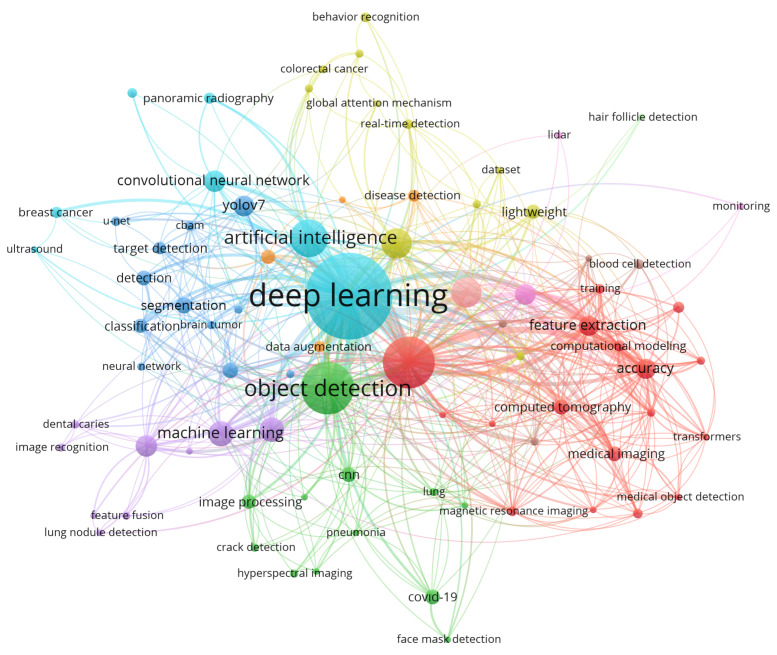
YOLO health bibliometric.

A bibliometric network analysis was performed on the articles indexed on the Web of Science database with the inclusion of the YOLO and health-related keywords in the abstract field of the articles. Following the initial search, the output was then manually screened to remove studies that involve search applications outside the field of human health or studies that involve applications of YOLO in other areas such as the field of agriculture, which involve other terms or concepts for “health,” for instance, the health of plants or animals, which form a significant proportion of other applications of YOLO. Figure 9 below indicates the important application areas covered under the purview of YOLO applications in the field of health-based systems using the YOLO architectures.

A keyword co-occurrence analysis was performed to identify the theme in the collected research. To that effect, both the occurrence and overall link strength of the keywords in a bibliometric network were determined. Looking at Table 5, there emerges a clear theme that revolves around terms such as “deep learning,” “object detection,” and “computer vision.” This is an indicator that such terms form a central theme associated with technological trends that underpin research in this theme. Additionally, an understanding can be derived regarding the overall strength and themes in research that emerges in conjunction with YOLO and healthcare themes.

## 5. Applications of YOLO in Healthcare Systems

The YOLO family of object detection algorithms has been widely explored in healthcare due to its real-time performance and high detection accuracy. In this section, we categorize and analyze the major application areas of YOLO in healthcare systems based on the current literature.

### 5.1. Medical Imaging (X-Ray, CT, MRI)

YOLO models have been extensively used in medical imaging applications owing to their capability to quickly detect and localize abnormal areas in complex anatomical structures. In radiography, specifically for chest and bone X-ray images, YOLO models like YOLOv3 and YOLOv5 have shown excellent results in detecting pulmonary infections like pneumonia and tuberculosis, as well as bone fractures. In Computed Tomography (CT), YOLO models are commonly used in applications such as pulmonary nodule detection and intracranial hemorrhage localization, where the efficient extraction of spatial features and fast processing are critical. While there are relatively fewer applications of YOLO models in Magnetic Resonance Imaging (MRI) modalities, promising results have been achieved for brain tumor localization and anatomical structure segmentation, particularly when YOLOv5 models are fine-tuned on medical dataset-specific data.

**Figure 10:**
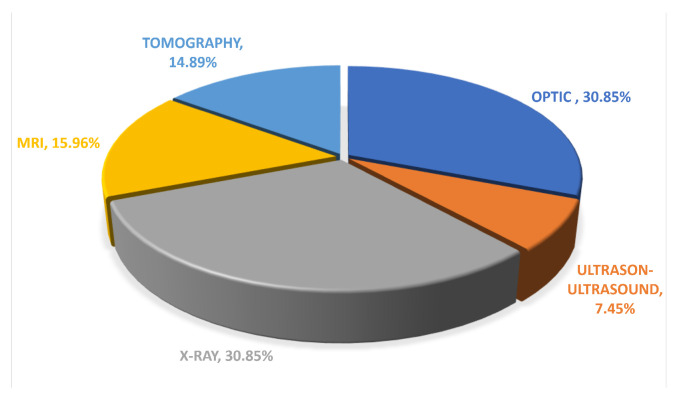
Distribution of medical imaging modalities used in YOLO-based healthcare applications.

Due to the architectural structure of the model and the real-time object detection feature, the primary usage of the YOLO model is related to multimodal images of the medical kind, which can be commonly observed in clinical settings. Figure 10 shows the break down of the usage of the YOLO model as per the type of medical images. It can be seen that X-ray images [61,66,67] as well as optical images [65,68,69] are the most commonly used, which are 29 in number each.

Magnetic Resonance Imaging (MRI) [70,71,72], with 15 studies, and Computed Tomography (CT)/tomography [49,73,74], with 14 studies, also make up a substantial number of studies. This can be associated with the rising interest in exploring YOLO models in cross-sectional and volumetric imaging modalities, which usually involve slice evaluation or two-dimensional projections. On the other hand, ultrasound studies have been investigated to a limited extent with only seven studies, and this can be considered due to its nature of high speckle noise, poor contrast, and strong operator dependence, making it difficult to investigate and detect objects in real-time appropriately. In any case, the graph makes it clear that YOLO models have yet to be fully explored in imaging modalities with well-defined spatial features and that its application in the other modality type remains a new and promising area of research.

### 5.2. Disease Detection (e.g., Cancer, COVID-19)

Beyond modality-specific applications, YOLO-based models are also essential in automated disease diagnosis for their ability to perform precise spatial regression and real-time processing. YOLO-based models have been widely employed for the detection of pathological features like skin cancer lesions in dermoscopic images, breast cancer in mammography images, and lung pathologies in CT scans. In the context of the COVID-19 pandemic, YOLOv3 and YOLOv4 models have received considerable attention for their ability to detect infection patterns in chest X-rays and CT scans. Their real-time processing ability makes them ideal for time-critical medical settings. To improve the diagnostic accuracy of these models, attention mechanisms, multi-scale feature extraction, and hybrid models have been employed in several studies.

**Figure 11:**
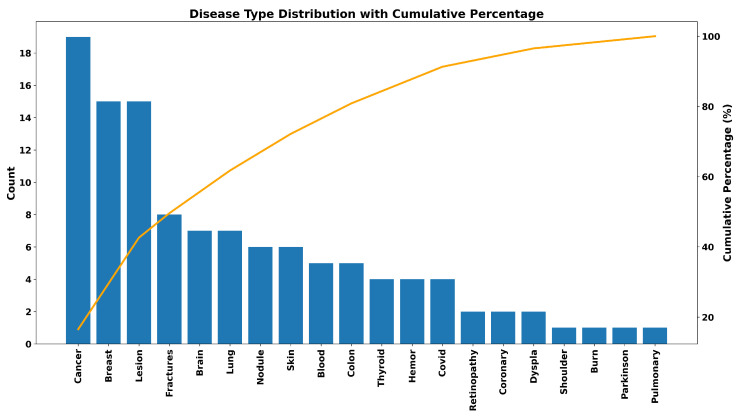
Overview of disease categories detected using YOLO-based models across healthcare imaging modalities.

Figure 11 shows the type of disease covered by YOLO detection models in medical applications and their cumulative percentage. A considerable number of studies in the literature pertain to cancer research [66,75,76,77,78]. Breast conditions [66,75,76,77,78] and overall lesion detection [79,80] follow suit, pointing towards the early diagnosis and detection of pathological features in medical images by YOLO models. Musculoskeletal ailments such as fractures [81,82,83] and other organ-specific ailments including those related to the brain [58,84,85], lungs [28,41,50], skin [86,87,88], and colon [56,89,90] also make up a substantial number of studies. On the other hand, diseases such as retinopathy [31,91], coronary conditions [92,93], COVID-19 [94,95,96], thyroid diseases [62,97,98,99], and hematological disorders emerge moderately, while less common applications such as burn evaluation, Parkinson’s disease [30], pulmonary diseases [100], and shoulder pathologies [101] emerge to be less prominent. In general, it has been observed from the cumulative graph that very few medical categories have made a majority of use of YOLO-based algorithms, and this indicates that most of the ongoing research work has already been directed towards clinically impactful and imaging.

### 5.3. Surgical Assistance and Navigation

YOLO models and architectures are being progressively utilized within the operating room setting for further improvement in surgical accuracy, safety, and overall operating room efficiency. Models and techniques, including YOLOv5 and YOLOv7, have been utilized for real-time surgical instrument detection and tracking, which helps in the automatic documentation of surgery and provides in-depth analysis and assistance for robotic or computer-assisted surgery. In the context of laparoscopic and endoscopic surgery, YOLO models have been utilized for anatomical structure recognition and the subsequent identification of anatomical landmarks within the operating room setting, which helps in avoiding the possibility of damaging critical areas. Smaller YOLO models, including YOLOv5n, have also been utilized for embedded systems with the specific requirement of providing real-time visual cues for surgeons, particularly for minimally invasive systems.

### 5.4. Patient Monitoring and Safety

A key benefit offered by YOLO-based systems for monitoring patients is their ability to greatly improve situational awareness in a healthcare setting by continuously analyzing patients’ behavior in real time using visual data. In the context of a fall detection system, for instance, a trained YOLO algorithm is capable of recognizing a falling position, abnormal position, or rapid movement from image streams to instantly notify healthcare professionals to respond to a situation. By combining the capabilities of pose estimation, YOLO can also assist in a comprehensive analysis of patients’ movement and position, which can help observe patients bedridden in a particular ward, detect early warning signs of discomfort or distress, and confirm patients’ proper positioning during their post-operative period. In a senior care setting, for that matter, systems based on YOLO can also assess long-term movement data to recognize deviations in normal activity and automatically send notifications for potential falls, wandering, or emergencies.

### 5.5. Smart Hospital Systems (e.g., Fall Detection, PPE Compliance)

Within the wider ecosystem of smart healthcare infrastructure, the role of YOLO in enhancing operational safety and efficiency in medical settings is quite substantial. During pandemics like the current COVID-19, there is massive use of YOLO models in the monitoring of PPE use, automatically identifying the use of masks, gowns, and face shields among medical staff in an effort to contain the spread of the pandemic. Additionally, YOLO-powered surveillance systems are also employed in intrusion and zone violation alerts, allowing for the immediate detection of unauthorized access to restricted zones and monitoring crowd density in high-risk settings to maintain safe standards. Moreover, critical asset monitoring systems developed with YOLO technology enable the identification and tracking of critical medical assets like defibrillators and wheelchairs, resulting in a reduction in the loss and optimization in the use of these resources. All these applications pertain to real-time edge technology platforms such as NVIDIA Nano, Raspberry Pi, where the high processing speed and relatively low computation needs in the graph constructed in YOLO are ideal for implementing in medical settings on an ongoing basis.

## 6. Performance Evaluation

Though various deep learning architectures have been developed for medical image analysis tasks, this review is deliberately conducted on the YOLO series of object detection models. The justification for this specific scope of work is based on the popularity of the YOLO series in real-time healthcare applications and its unique trade-off between object detection accuracy and computational complexity. Through this specific scope of work on the evolution of the YOLO series from version v1 to v12, this research aims to provide a more in-depth analysis on the topic.

The presentation and synthesis of evaluation metrics are critical in a systematic review of YOLO-based healthcare applications, as they enable objective comparison across studies and architectural variants. Metrics such as mean average precision (mAP), precision, recall (sensitivity), specificity, F1-score, Area Under the Curve (AUC), and inference time provide standardized indicators of diagnostic reliability, localization performance, and computational feasibility. By systematically analyzing the reported metrics, this review not only summarizes performance outcomes but also highlights inconsistencies in evaluation practices and underscores the need for more standardized, clinically meaningful benchmarking frameworks in medical object detection research. In the evaluated literature, different evaluation criteria have been used to assess the performances of the YOLO architecture in healthcare applications. These criteria comprise elements described below.

### 6.1. Accuracy

In its classical form, accuracy is defined as the proportion of correctly classified samples over the total number of samples as depicted in Equation (1):

(1) $$Accuracy=\frac{TP+TN}{TP+TN+FP+FN}$$

In the equation above, TP denotes True Positive, TN denotes True Negative, FP denotes False Positive, and FN denotes False Negative. However, the fact is that the YOLO is a dense prediction algorithm because it breaks down an image into several grid cells such that for every image, multiple bounding boxes and class predictions are made. Therefore, the true negatives are so high that accuracy can easily be inflated. Moreover, it is possible to develop an algorithm with high accuracy which struggles to detect some critical objects in the image. As such, it is important to exercise caution when accuracy is used as the main yardstick for the evaluation of an object detection algorithm like YOLO.

For instance, the study on Breast Lesion Detection and Classification indicated a total detection accuracy of 99.17% on the DDSM dataset [102], while the study on Rib Fracture Detection indicated an accuracy level of 97% [103]. In a similar manner, the study on Automatic Dermatology Detection indicated an accuracy level of 90% for classification of melanoma [104].

### 6.2. Intersection over Union (IoU)

Intersection over Union (IoU) is a geometrical evaluation parameter that assesses the overlap between the predicted bounding box and the actual bounding box for an object in an image. This parameter helps to determine the ability of the model to localize an object within an image accurately. This is calculated using the equation below:

(2) $$IoU=\frac{AreaofOverlap}{AreaofUnion}$$

where Area of Overlap = intersection area between Bbb, Gbb, Area of Union = total area covered by Bbb, Gbb. Usually, if Intersection over Union ratio satisfies a certain threshold, that threshold value settles generally at 0.5, and a recognition system can be considered to be working correctly. Take as an example a Lung Cancer Cytological Segmentation system from [105], which achieved a Mean Intersection over Union (MIoU) value on test dataset of 0.70, and a Cerebral Hemorrhage Volume Measurement system from [27] where as a major achievement, MIoU was attained as 87.1%.

### 6.3. Mean Average Precision (mAP)

For a single class of objects, average precision (AP) calculates how well a model performs on various confidence levels. Mean average precision (mAP), on the other hand, is recognized as a standard performance metric that has long been used for the assessment of various object recognition models, including YOLO, as per Equation (3) as follows:

(3) $$mAP=\frac{1}{N}\sum_{i=1}^{N}AP_{i}$$

where *N* = number of classes and *AP_i_* is the average precision of class *i*.

This criterion estimates the accuracy and location of objects by the model. It calculates the average precision for all classes and at different levels of IoU. The different forms are mAP@0.5 and mAP@[0.5:0.95]. For instance, in the ulcers detection model in reference [106], an average precision of 76.9% was reported using YOLOv5, while in Medical Microscopic Smear Detection in reference [107], the proposed model attained an average precision of 92.5% in the BCCD dataset. In addition, in the developed Smart Bone-Age Assessment System [108], the authors proposed that the YOLO-DCFE structure reached an mAP@0.75 value as 0.995 and an mAP@0.5:0.95 value as 0.732.

### 6.4. Precision

In YOLO-based object detection, precision measures the reliability of the model’s positive predictions—that is, how many of the detected objects are actually correct. Its formulation is defined in Equation (4),

(4) $$\mathrm{Precision}=\frac{TP}{TP+FP}$$

In this regard, TP or True Positive refers to the accurate identification of objects by the model, which means the classification and localization of the object meet the required criteria of classification and localization. On the contrary, FP or False Positive refers to the incorrect identifications made by the model, which could be due to improper classification or an inappropriate level of localization. Therefore, precision with a high value means there is a minimal rate of false alarm and correct predictions made by the model.

In the study of brain tumor lesions, the authors were able to show that the precision of the developed and improved YOLOv8 algorithm attained a precision of 95.4% [109]. Similarly, in the study of blood cell detection, the developed algorithm attained a precision of 86.90% [110].

### 6.5. Recall

Recall: Recall checks the capability of the YOLO algorithm in detecting the appropriate number of objects inside the image. Recall measures the exact number of ground truths detected by the detector. Recall is represented as in Equation (5).

(5) $$\mathrm{Recall}=\frac{TP}{TP+FN}$$

In this respect, the True Positive (TP) represent things that are identified correctly by the model, whereas the False Negative (FN) stands for instances that exist in the ground truth but are not detected during the process of detection. A high recall measure thereby ensures that a large majority of relevant instances are identified by the model with less false misses, which is highly important in safety-critical and healthcare-oriented systems, as false misses can cause serious consequences.

In conventional YOLO-based object detection models, for a detection to be regarded as a True Positive, there are two requirements that must be fulfilled simultaneously: first, the predicted class label must match the actual/ground truth class label, and second, there must be a sufficient overlap between the predicted bounding box and the respective actual/ground truth box annotation, usually indicated by a certain IoU value. In most contemporary YOLO-based research work that focused on healthcare applications, high recall scores were obtained for tumor detection tasks. Specifically, for tumor detection tasks, a high recall of 95.2% was obtained by the Enhanced TumorNet model that employed a U-Net structure and a YOLOv8s model for brain tumor segmentation and analysis purposes [111]. Another study by Aydemir et al. introduced a model for the detection and classification of breast masses from mammographic images using the CBIS-DDSM dataset and a transfer learning approach, and it reported a high recall of 0.774 for reliable detection of breast lesions, which is a guaranteed indicator of good detection performance for breast lesions in mammographic images [112].

### 6.6. F1-Score

The F1-score is the harmonic mean of precision and recall, as shown in Equation (6). It gives a measure of balancing both false positives and false negatives in the output of a model or system. In a YOLO environment, this measure can be used to evaluate the performance of detected results based on confidence and IoU thresholds.

(6) $$F1\text{-}\mathrm{Score}=2\times \frac{Precision\times Recall}{Precision+Recall}$$

YOLO models generate multiple object detections, each with an associated confidence score. To compute the F1-score, an IoU threshold—for example, 0.5—is fixed in advance, followed by the choice of a confidence threshold that filters the detections. Based on the remaining predictions, values of precision and recall are calculated, and from these, the F1-score can be calculated. In practice, this process is repeated across a range of confidence thresholds, and the maximum F1-score is commonly reported, as it represents the optimal balance between precision and recall for the given detection model.

F1-score is widely being used in healthcare-oriented YOLO studies to assess the balance between precision and recall. In the lumbar intervertebral disc degeneration analysis based on the Pfirrmann grading system, the F1-score of the model is 97.77% for Grade IV in the training set and 95% in both the testing and external validation sets [60]. Similarly, a tracheal lesion detection model called BrYOLO-Mamba has reported an F1-score of 91.7% on the hybrid bronchoscopy dataset with superior performance compared to state-of-the-art deep models based on CNN and Transformer models [64].

### 6.7. Inference Time/Frames per Second (FPS)

Inference time, a critical metric for real-time healthcare applications such as surgical navigation and patient monitoring, it indicates how fast the model can process input images. In a typical experimental setup that characterizes inference performance, first, a YOLO model is loaded and configured from memory for deployment. Then, under specific experimental conditions, such as fixed input resolution, batch size, and configuration of hardware, a batch of *N* input images or video frames is processed using the model. In this process, the total time required to complete the predictions for all *N* samples is measured with high accuracy, usually excluding the overhead caused by loading data and pre-processing. Finally, the inference speed is expressed in the unit of FPS (Frames Per Second), which is obtained as a ratio of the number of processed frames to the total time taken for inference and gives a simple, hardware-dependent measure of the model’s runtime efficiency.

**Figure 12:**
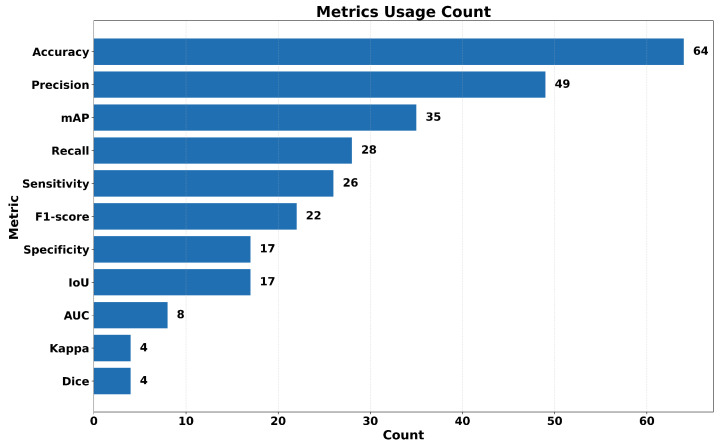
Used performance metrics.

These two measures are commonly gauged together to form a balanced perspective, depending upon the constraints of the healthcare task. The combination of these measures, as presented in the literature, varies. Figure 12 illustrates the proportion of the distribution of the measures of evaluation used in the studies, which dominate the measures of accuracy, precision, recall, and mAP. The significantly larger proportion of the use of the measure of accuracy, as compared to the other measures, indicates the commonly used perspective of gauging the performance measurements of the measures. Additionally, the proportion of precision–recall axis and mAP measures illustrates the necessity of these measures in clinical and robotic tasks, which involve unbalanced class distribution, multi-object detection, and error measures.

Additionally, more application-centric or task-aligned criteria such as training time, inference time, FPS, dice, AUC, specificity, and IoU are also considered to possess a fairly low level of usage. This implies that performance measurement in the literature primarily targets output validation or verification, whereas operational criteria related to real-time, computational expense, or system efficiency are of less importance. On the other hand, the fairly low level of usage of criteria such as AUC, specificity, or IoU also implies a certain level of variation in this domain as well, because criteria relating to specific tasks such as segmentation or classification may apply in specific ways depending on the problem domain being addressed.

### 6.8. Benchmark Datasets Used in Studies

Healthcare research based on YOLO normally makes use of publicly available datasets in combination with those collected from specific institutions. Commonly used datasets include FracAtlas, which is a musculoskeletal X-ray scans specifically curated for bone fracture classification [113]; the CEM dataset, which is a multi-institutional collection of Contrast-Enhanced Mammography (CEM) scans for breast cancer detection [52]; NIH ChestX-ray14, widely adopted for the detection of conditions like pneumonia [59,95], tuberculosis, and COVID-19; the SIIM-ACR Pneumothorax Dataset, commonly adopted in YOLOv4- and YOLOv5-based lung abnormality detection research; and LUNA16, a standard benchmarking dataset used in CT scan pulmonary nodule detection research [41,83]. With regards to breast imaging, the Breast Cancer Digital Repository is one of the most adopted datasets for tumor detection in mammograms, while the datasets of the EndoVis Challenge support surgical tool detection and tracking tasks in laparoscopic procedures. Adapted subsets of more general-purpose datasets such as PASCAL VOC [12] and COCO are sometimes repurposed with medical annotations to create benchmark datasets. In the case of more specialist applications—such as dental imaging, endoscopy, or monitoring personal protective equipment compliance—researchers often build their own custom-labeled datasets in collaboration with medical institutions.

### 6.9. Comparative Overview of YOLO-Based Architectures in Healthcare Domains

Table 6 presents a comparative overview of YOLO-based architectures applied across diverse healthcare domains, highlighting their task-specific enhancements and reported performance outcomes. The table demonstrates that modern variants such as YOLO-NAS, YOLOv11, and hybrid YOLO frameworks consistently achieve high accuracy, mAP, and F1-scores while maintaining real-time processing capabilities. In particular, attention-enhanced, transformer-integrated, and lightweight adaptations show notable improvements in detecting small, complex, or overlapping medical findings. Overall, the comparative analysis illustrates the adaptability and scalability of YOLO models in addressing heterogeneous diagnostic challenges across clinical settings.

## 7. Discussions and Challenges

Although the applications of YOLO-based object detection systems have significantly developed in healthcare, a lot of challenges and unaddressed research gaps still exist before smooth integration into clinical applications can be realized. This section assesses the main challenges identified from the literature review based on all the studies analyzed in the course of this work.

### 7.1. Clinical Validation and External Generalizability

Although YOLO-based architectures have proven to achieve promising performance metrics, such as mAP, accuracy, and sensitivity, in controlled experiments, it is vital to perform clinical validation of these models before they can be translated to real-world clinical practice. The majority of the studies reviewed in this article used retrospective databases, which were collected from a single source. This therefore limits the generalizability of the results. In clinical diagnostics, model robustness with respect to different imaging modalities, patient demographics, and healthcare institutions is of paramount importance. The imaging modalities used in clinical diagnostics vary in terms of the manufacturer of the imaging device. The imaging protocol also varies significantly. These factors can impact the performance of the model. Therefore, it is vital to perform prospective validation of YOLO-based detection systems to ensure that they perform consistently in different healthcare institutions.

### 7.2. Limitations

There was no quantitative weighting method employed in the comparison of systematic reviews with non-systematic reviews. The comparison was carried out qualitatively with regards to their methodological structure, scope, and database coverage. Future studies can include normalized bibliometric measures in their analysis.

As an additional limitation, it is important to note that the comparisons of performance highlighted in this review are based on structured narrative synthesis rather than actual quantitative analysis. This is because of the heterogeneous nature of imaging modalities, disease types, dataset sizes, conditions of class imbalance, etc. Thus, it is pertinent to mention that clinically relevant factors such as sensitivity, specificity, false positive/false negative implications, etc., have also been highlighted in the context of technical performance. However, it is noteworthy that high performance at the benchmarking level does not necessarily imply clinical readiness.

Effective real-world application of YOLO models in the health sector demands high generalizability in varied medical environments rather than optimal performance in the same training set. Many authors have noted significant drops in performance when transferring models into other hospitals, machines, protocols, or even patients when the environments of acquisition vary. To deal with these issues, the literature suggests the use of domain adaptation, training models using data involving multiple medical centers and diverse demographics, ensemble learning, and fine-tuning YOLO models pre-trained for foreign environments. Yet, the improvement of generalizability still represents an important challenge in this field since adaptability is required for the safe employment of models in the medical sector. There are a number of important considerations that must be taken into account when implementing AI systems in a real-world medical setting such as regulatory pathways that must be approved by government agencies such as the FDA and require a clear demonstration of clinical efficacy and safety before use, or data privacy which is also a very important issue, requiring a high level of compliance with regulations. Additionally, new approaches such as federated learning provide promising solutions for secure data use. Algorithmic bias is also a major concern, as AI systems may perform suboptimally on minority patient groups, emphasizing the need for diverse and representative training data. There are also accountability issues, which are complex from a legal and ethical perspective, especially regarding liability for an incorrect AI-assisted diagnosis. Lastly, patient safety is a critical concern, as false positives can cause unnecessary procedures and patient anxiety, while false negatives can cause delayed diagnoses and serious harm.

### 7.3. Ethical Constraints and Thrust

Clinical adoption of YOLO-based AI systems into healthcare relies not only on technical performance but also on meeting strict regulatory and ethical requirements. Regulatory approval frameworks, such as FDA clearance via the Software as a Medical Device pathway in the United States, and CE marking according to the European Medical Device Regulation, need to be clearly demonstrated with clinical effectiveness, acceptable levels of safety, and comprehensive risk analyses using real patient data. Ethical considerations also extend to patient privacy and data protection—considering, for example, GDPR and HIPAA—informed consent, liability resulting from incorrect AI-assisted decisions, and mitigation of algorithmic bias due to nonrepresentative training data. In this regard, the literature focuses on early engagement with regulatory bodies, employment of privacy-preserving learning methods, transparent reporting of model performance across diverse populations, and well-defined responsibility frameworks. Cumulatively, these regulatory and ethical issues are vital to turning YOLO-based systems from research prototypes into clinically reliable tools.

This implies that even with high accuracy, trust needs to be established within healthcare professionals for YOLO-based artificial intelligence systems if they are to find a place as trusted decision-support tools. This is in addition to technical reliability and quick integration into clinical workflows. Several concerns arise from risks related to false positives leading to many unnecessary examinations, increased costs, and patient anxiety, and of false negatives, which delay treatments and may therefore result in severe or irreversible outcomes. Sometimes, the lack of clinical interpretability may also make clinicians discouraged from relying on model outputs due to unclear reasoning behind the output. Trust should be built in a very careful and rigorous manner since diagnostic errors during patient care may not only be costly but also life-threatening. Thus, the literature points out that it is necessary to conduct comprehensive clinical validation studies across multiple centers, positioning AI systems as supportive tools within a human–AI collaboration framework, and implementing explainability mechanisms that highlight decision-relevant image regions.

### 7.4. Clinical Interpretability and Explainability

Interpretability in the context of diagnostic medicine, however, cannot be seen as optional; rather, it becomes a necessity to establish trust. While YOLO models do produce bounding boxes and confidence levels, these factors may not be enough to explain why a lesion or abnormality was detected. What becomes a necessity in such scenarios is to have a transparent reasoning system that helps the clinician validate whether the model is indeed looking at meaningful areas of the image or just seeing irrelevant features. Explainability techniques such as Grad-CAM, saliency maps, and attention visualization do exist to aid such requirements. However, these techniques have not been consistently employed in YOLO-based research in the healthcare domain. The incorporation of explainable AI techniques into YOLO models becomes a necessity to ensure clinician confidence in the model.

### 7.5. Inconsistent Evaluation Metrics Across Studies

The evaluation of the performance of YOLO-based models with regards to healthcare applications has shown a high degree of variability due to the use of different metrics, datasets, and experimental protocols. Even though mAP, precision, recall, IoU, FPS, and inference time are commonly reported, their inconsistent application, together with the lack of standardized healthcare benchmarks, differences in hardware and software settings, and private datasets, limits comparability. For this reason, the literature underlines the importance of shared benchmark datasets, unified evaluation protocols, transparent reporting of experimental settings, and independent validation that would allow for reliable performance assessment, enabling clinical adoption for YOLO-based methods.

### 7.6. Computational Demands and Edge Deployment Challenges

Real-time healthcare object detection using YOLO-based applications has many computational challenges, mainly for edge devices with low resources, such as Jetson Nano, Raspberry Pi, and embedded NVIDIA platforms. The new versions of YOLO with higher accuracy increase the depth of the architecture along with attention mechanisms, hugely amplifying GPU, memory, and bandwidth usage. The complexity is further enhanced in real-time healthcare applications due to several megabytes of medical images, like radiology scans in high-resolution or gigapixel pathology slides. In an edge environment, specific limitations exist regarding computational resources, limited energy budgets, thermal issues, and ultra-low-latency requirements within surgical assistance or patient monitoring applications. Solutions that can mitigate this include model compression through pruning, quantization, and distillation; resolution-aware or patch-based processing; hybrid architectures on edge–cloud; utilization of off-the-shelf hardware accelerators such as FPGAs, TPUs, and dedicated AI chips for enabling an efficient and reliable way to run YOLO-based inference on healthcare systems.

### 7.7. Integration Challenges with Clinical Workflows

The successful translation of the robust laboratory results achieved by YOLO-powered AI solutions in a laboratory setting to a clinical setting is largely contingent on their effective integration in current clinical settings and applications. Accuracy and rate of processing are not adequate; these solutions need to work in harmony in the ordinary setting of clinicians as a component of Hospital Information Systems. Key integration challenges include technical compatibility with PACS and EHR systems that often rely on proprietary standards, real-time data flow to ensure millisecond-level responsiveness in time-critical settings, user-friendly interfaces that can be operated without technical expertise, ongoing maintenance and updates within busy hospital IT environments, and robust cybersecurity measures to protect sensitive healthcare data during network-based transmission. Addressing these issues is essential for the sustainable and safe clinical adoption of YOLO-based systems.

## 8. Future Directions

As YOLO architecture continues to evolve, the role of these models in healthcare is expected to expand significantly. Future research and development efforts should focus on enhancing adaptability, interpretability, and trustworthiness.

### 8.1. Potential Improvements in YOLO for Healthcare

Today, robust object detection models such as YOLO require large, heterogeneous, and distributed datasets for use in healthcare; due to patient privacy concerns, these data are often hard to collect in one place. Federated learning (FL) closes this gap by enabling several organizations to collaborate on a common global model without actually having to share data. FL is effective for such tasks as the classification of medical images—for example, the detection of brain tumors and COVID-19. DPCOVID study proposed the usage of FL in conjunction with differential privacy—DP-SGD—for COVID-19 detection on chest X-rays [114]. Li et al. used FL on the segmentation of brain tumors, ensuring the security of patients’ data [115]. Furthermore, Yang and coauthors presented MixFedGAN, a framework that involves a dynamic fusion and information distillation method within FL while dealing with heterogeneous and limited-label data [116]. Koutsoubis et al. also introduced uncertainty estimation to FL, enhancing model confidence in medical images [117]. Finally, the work in [118] developed the system which combined high accuracy and patient privacy in brain tumor detection via MRI using EfficientNet-B0 with FL.

The integration of real-time perception models such as YOLO with federated learning in the medical field presents many opportunities in terms of both efficiency and privacy issues. FL presents opportunities for the deployment of YOLO in various hospital and clinical settings without the need for data centralization. This is an appropriate approach that will enable the training of the model on data with various devices and anatomical structures. However, the success of this approach is dependent on addressing the issues associated with data heterogeneity (non-IID data), communication delays, and trade-offs on privacy risks (Differential Privacy, for instance). Finally, in this field, federated learning approaches customized to the requirements of YOLO, such as uncertainty-weighted aggregation with methods related to information distillation for local model update, are very promising future research directions, especially for medical applications with perception in the domain of real-time key point detection in surgery.

### 8.2. Integration with Other AI Models (e.g., Transformers, Hybrid Models)

YOLO perception models are being very rapidly assimilated for more powerful and specialized tasks using transfer learning. They are also hybridized with other models (ResNet, EfficientNet, U-Net/ResUNet, Transformer blocks, and others) [119] to further enhance the extraction of features and spatial accuracy [120]. For instance, in field-level industrial detection experiments, by exploiting the kernels of YLO that are pre-trained on the COCO or ImageNet task and adapting them for domain-level features, i the scarcity of data was lessened significantly and also the inference time was kept feasible. By being coupled with ResNet or EfficientNet models to further raise the channel/spatial level of representation, marked improvements are delivered, mainly for distant perception and engineering level inspections.

The most effective approaches for hybrid designs usually involve either one or a combination of the first two approaches: (1) Backbone transfer/training or “swapping”—initially maintaining YOLO’s “head–neck” network structure along with strong, transfer-trained classifiers or feature extractors (ResNet, EfficientNet, Swin/Transformer pre-trained layers); (2) Task-driven fusion modules—incorporating YOLO’s detection module with the use of U-Net or “Attention” modules for jointly focusing on both local (pixel-level) or contour information alongside global views. For instance, in medical image analysis tasks, YOLO-U-Net hybrids have succeeded in detection as well as in accurate localization of detailed structures like keypoints in surgical procedures or tumor boundaries, while in RS or industrial defect detection studies, YOLO variants that incorporated transfer learning for fine-tuning likewise achieved superior generalization capabilities than fully new “//scratch”-trained networks. For future research, thus, (i) “distillation techniques” enhancing information flow between backbone and “head” networks, (ii) multi-task joint training for detection and “pixel-wise” image segmentation tasks, and (iii) “domain adaptation” of transfer-learned feature representations in hybrid CNN-Transformer networks are key.

### 8.3. Role in Real-Time and Edge-Based Healthcare Systems

More recently, lightweight YOLO frameworks for object detection have been vigorously explored for real-time processing on resource-constrained devices. Edge-YOLO [121] achieved a 71.6% reduction in model size and a 44.4% improvement in speed for infrared target detection by introducing ShuffleBlock modules and content-aware CAU-Lite upsampling. LEAF-YOLO [122] obtained >30 FPS for real-time processing on the Jetson AGX Xavier through multi-scale feature extraction and parameter-effective modules for small object detection. CFIS-YOLO [123] improved mAP by 4% with a 17.3% decrease in energy consumption on the SOPHON BM1684X for industrial wood defect detection by employing CARAFE upsampling and FasterBlock modules. Edge-Optimized Lightweight YOLO [124] executed at 34.2 FPS on the Jetson TX2 board with 1.9 million parameters for SAR image detection via an efficient backbone with an inverted bottleneck module structure with fast pyramid fusion.

These studies highlight the importance of a holistic architectural optimization that balances both accuracy and speed requirements, despite challenges such as scale variability, complex backgrounds, low contrast, and data scarcity encountered in diverse domains (infrared imaging, unmanned aerial vehicles, industrial defect detection, SAR imagery). The developed lightweight YOLO models deliver high mAP values with low latency by combining feature extraction, multi-scale information fusion, and prediction headers. This enables high-accuracy and energy-efficient solutions in scenarios requiring real-time object detection at edge devices, such as autonomous vehicles, industrial quality control, security monitoring, and disaster management. In healthcare, particularly in edge devices such as wearable sensors, smart cameras, or portable diagnostic devices, such optimized architectures provide a low-latency, energy-efficient foundation for real-time analysis of patient movements, emergency symptoms, or medical images as used in [125]. This enables secure on-site processing of continuous data streams, reducing network traffic and enabling rapid clinical decision support while preserving privacy.

These studies also underscore the need for the overall optimization of architectural design catering to the requirements of accuracy as well as speed, despite the challenges posed by variability in size, complicated backgrounds, contrast scarcity, and dataset unavailability in various application domains (unmanned aerial vehicles, infrared imaging, industrial defect detection, and SAR images). The proposed YOLO models achieve high mAP and high speed with minimal latency while incorporating features extraction, multiscale fusion, and prediction headers. These models cater to high-accuracy and energy-efficient applications with real-time object detection requirements in edge devices in the automotive industry for self-driving cars, industrial quality inspection and control in industries, security monitoring systems, and disaster response applications. In medical applications, in edge devices used in wearable sensors, smart cameras, or handheld diagnosis units in the medical sector, such design-optimized versions create a robust edge infrastructure with minimal latency and energy requirements for real-time processing of patient motion, emergency symptoms recognition, or medical image processing at the edge.

### 8.4. Emerging Research Directions: Efficiency, Deployment, and DataModel Trade-Offs

To promote advancement in the area, we stress the importance of a three-fold research focus. First, the community needs FLOPs and parameter-level benchmarking of YOLO variants in the context of medical imaging applications to establish a baseline for performance. Second, researchers need to perform thorough assessments of edge device deployment constraints, targeting real-world considerations such as latency, memory requirements, and energy efficiency. Third, there is a pressing need for empirical research that explores the relationship between dataset size, annotation density, and the choice of the best-suited YOLO variant for a particular medical application.

## 9. Conclusions

The results of this systematic review, based on 93 PRISMA-screened studies, suggest that YOLO architectures have evolved into a popular detection solution in the healthcare domain, especially in the context of radiological and optical imaging. The frequency of model usage analysis clearly shows that YOLOv5 and YOLOv8 are the most widely used models, comprising close to 90% of the studies reviewed, indicating a clear preference for models that provide a good trade-off between detection accuracy and computational complexity. This is not surprising, given the popularity of X-ray and optical imaging modalities, which comprised the largest proportion of studies reviewed. The trend towards YOLOv9–YOLOv12 models also indicates a clear shift towards models that focus on better feature aggregation, attention, and efficiency, although their usage in clinical studies is still relatively low.

Across application domains, YOLO-based models are most frequently employed in cancer detection, lesion localization, fracture identification, and pulmonary disease analysis, with cancer-related studies forming one of the largest thematic clusters. However, the analysis of the challenge reveals some shortcomings. The most common problems are the limitations of computational efficiency (36.6%), the difficulty of fine-grained lesion localization (35.5%), the limitations of the dataset (32.3%), and multimodal complexity (29.0%). Many reviewed studies report high detection performance within their respective experimental settings. To overcome these difficulties, recent research has started using transfer learning, multi-scale feature fusion, hybrid CNN-Transformer models, and lightweight models to improve robustness and feasibility.

Beyond technical performance, the findings emphasize that high accuracy and fast inference alone are insufficient for real-world diagnostic adoption. Critical challenges remain related to model interpretability, explainability, clinical validation, ethical compliance, and seamless integration with existing hospital information and picture archiving systems. These considerations underscore the importance of clinically transparent and trustworthy AI systems, where YOLO-based models function as decision-support tools rather than opaque black-box solutions. Future research directions therefore include the development of explainable YOLO-based diagnostic models, standardized benchmarking across heterogeneous medical datasets, multi-center clinical evaluations, and algorithmic designs aligned with clinical reasoning, regulatory requirements, and patient safety standards.

In conclusion, YOLO architectures have established themselves as foundational components of real-time computer vision for medical diagnostics, offering an effective balance between speed and detection accuracy across a wide range of clinical applications. By presenting consolidated scientific evidence, identifying dominant architectural trends, and outlining unresolved diagnostic challenges, this systematic review serves as a valuable reference for researchers, clinicians, and healthcare stakeholders seeking to design, evaluate, and deploy YOLO-based diagnostic systems in next-generation healthcare environments.

## Figures and Tables

**Table 1:** Inclusion and exclusion criteria.

Inclusion Criteria	Exclusion Criteria
Peer-reviewed journal articles; Studies explicitly using YOLO or its variants (e.g., YOLOv3, YOLOv5, YOLOv11); Applications within healthcare contexts (e.g., medical imaging, patient monitoring, disease detection); Publications in English; Articles published between 2018 and 1 January 2026.	Studies not applying YOLO-based architectures; Papers outside the healthcare domain (e.g., agriculture, industrial or general surveillance); Review papers, editorials, white papers, or non-peer-reviewed sources; Articles without accessible full-text; Publications in language other than English; Publication before 2018; Studies focused on dentistry (excluded to maintain a focused scope).

**Table 2:** Study quality assessment framework across five domains.

Domain	Key Evaluation Criteria	Reporting Level (Scoring)	Interpretation
**Dataset Transparency**	Dataset source clearly stated; sample size reported; class distribution described	2 = Fully reported 1 = Partially reported 0 = Not reported	Evaluates reproducibility and adequacy of data representation
**Validation Strategy**	Clear train/validation/test split; use of cross-validation; external validation (if applicable)	2 = Robust validation (Cross Validation and/or external validation) 1 = Basic split only 0 = Unclear or inadequate validation	Assesses methodological rigor and reliability of performance claims
**Performance Reporting**	Standard metrics (mAP, sensitivity, specificity, precision, recall); multiple complementary metrics; confidence intervals reported	2 = Comprehensive metrics 1 = Standard metrics only 0 = Limited or unclear metrics	Measures transparency and statistical robustness of reported results
**Overfitting Assessment**	Absence of data leakage; justified augmentation strategy; external/generalization testing	2 = Explicit overfitting mitigation and generalization analysis 1 = Partial discussion 0 = No evidence provided	Evaluates model generalizability and robustness
**Clinical Relevance**	Addresses real diagnostic need; clinical interpretation of outputs; deployment considerations (workflow, edge devices, integration)	2 = Strong clinical integration 1 = Partial clinical discussion 0 = Purely technical study	Assesses practical applicability in healthcare environments

**Table 3:** Comparison of the previous reviews related to YOLO.

Ref, Year	Keywords	Used YOLO Models	Review Type	Focus Area	Year Span	Used Databases/Datasets
[17], 2024	YOLO, single stage detection, YOLOv10, YOLOv11, performance evaluation; deep neural network; real-time object detection	YOLOv1 to YOLOv11	Systematic	Evolution and benchmarks in healthcare, autonomous systems, and agriculture.	2015–2023	IEEEXplore, SpringerLink, CVPR, ICCV, ECCV
[18], 2025	Autonomous driving, object detection, YOLO algorithm, applications	YOLOv1 to YOLOv12	Non-systematic	Autonomous driving scenarios (vehicles, pedestrians, signs, lights, lane lines).	2017–2024	Science Direct, Web of Science, IEEEXplore
[19], 2025	Lightweight YOLO, Resource-constrained, SLR	YOLOv1 to YOLOv11	Systematic	Optimization for edge devices (pruning, quantization, KD).	2016–2024	MDPI, Springer, IEEE, Elsevier, Frontiers, Tech Science, SAGE, IET
[20], 2025	YOLO variants; real-time defect detection; fabric detection; deep learning in textiles; convolutional neural networks; textile industry; quality control	YOLO-v1 to YOLO-v11	Systematic	Automated quality control in textiles (tears, stains, holes).	N/A	N/A
[21], 2024	YOLO, healthcare applications, artificial intelligence, medical object detection, medical imaging, systematic review	YOLOv1 to YOLOv8	Systematic	Medical diagnostics (oncology, pathology, radiology, surgical tools).	2018–2023	PubMed
[22], 2023	YOLO, UAV, object detection, interdisciplinary, application	YOLOv1 to YOLOv7	Non-systematic	Aerial monitoring in engineering, agriculture, and rescue.	2017–2022	Web of Science, KCI, MEDLINE®, SciELO, CNKI
[23], 2023	industrial defect detection; object detection; smart manufacturing; quality inspection	YOLOv1 to YOLOv8	In-depth Review	Industrial surface defect detection.	2015–2022	N/A
[24], 2024	Deep learning, Images, Fruit detection, Computer vision, Transfer learning, Automation, Digital tools	YOLOv1 to YOLO-NAS	Systematic	Agricultural object recognition (crops, pests, diseases, animals).	2015–2024	Scopus

**Table 4:** Frequency of reported technical challenges and corresponding mitigation strategies in YOLO-based healthcare studies.

Challenges	Possible Solutions	Occurrence	Percentage
Limited or Imbalanced Training Data Availability [52,53,54]	Application of data augmentation strategies and transfer-learning–based model initialization to improve sample efficiency and mitigate class imbalance effects	30	32.3%
High Variability and Acquisition Noise in Medical Imaging Modalities [55,56,57]	Utilization of multi-scale representation learning and attention-driven feature refinement to enhance robustness against noise, resolution loss, and acquisition artifacts	9	9.7%
Multimodal Complexity and Heterogeneous Diagnostic Features [35,40,58]	Deployment of hybrid or ensemble deep-learning pipelines incorporating multimodal feature fusion (e.g., CNN–Transformer or dual-stream architectures)	27	29.0%
Domain Shift and Limited Generalization Across Imaging Conditions [36,59,60]	Transfer-learning regimes, domain-aware augmentation techniques, and transformer-based global feature modeling to improve cross-domain generalization performance	10	10.8%
Difficulty in Accurate Lesion Localization and Fine-Grained Segmentation [56,57,61]	Integration of advanced object-detection frameworks (YOLO variants), encoder–decoder segmentation architectures (e.g., U-Net), and multi-scale fusion mechanisms for improved spatial precision	33	35.5%
High False-Positive/False-Negative Rates in Automated Diagnostic Classification [29,57,62]	Adoption of attention-enhanced classification modules, hybrid CNN–Transformer decision models, and optimized task-specific loss functions to improve discriminative sensitivity and specificity	6	6.5%
Computational Efficiency Constraints in High-Resolution Image Analysis [63,64,65]	YOLO-based architectures and streamlined CNN backbones supported by loss-function optimizations and inference-time acceleration techniques	34	36.6%

**Table 5:** Keyword occurrences and total link strength.

ID	Keyword	Occurrences	Total Link Strength
1	deep learning	186	366
2	object detection	81	170
3	YOLO	79	210
4	artificial intelligence	47	89
5	computer vision	33	74
6	YOLOv8	33	78
7	machine learning	24	48
8	YOLOv5	23	46
9	attention mechanism	18	38
10	convolutional neural network	18	33
11	feature extraction	16	80

**Table 6:** Comparative analysis of YOLO models across healthcare systems.

Healthcare Domain—Specific Application	YOLO Models and Enhancements	Performance Metrics and Comparison
**Gastroenterology—Colorectal Polyp Detection**	**CE-YOLO:** Incorporates Partial Channel Cross-Stage Connections (PCST) and Dynamic Cross-Branch Fusion (DCBM) for highly efficient, lightweight processing [13]. **YOLOv8m:** Optimized anchor-free architecture [56]. **YOLOv3/v4:** Trained with negative samples to reduce false positives [14].	**CE-YOLO** achieved an F2 score of 86.14% and 85.24% recall, outperforming heavier models like YOLOv8 and YOLOv10 while using fewer parameters. **YOLOv8m** achieved 95.6% precision and a 92.4% F1-score, providing an excellent speed-accuracy balance. **YOLOv3/v4** with negative samples achieved an ultra-fast 100–122 FPS for real-time video processing.
**Neurological Systems—Brain Tumors and Hemorrhage**	**YOLO-NAS:** Neural Architecture Search for optimal multi-class tumor detection [58]. **Hybrid YOLOv8s + U-Net:** Fuses YOLOv8’s rapid localization with U-Net’s precise segmentation [111]. **YOLOv5x-GCB:** Uses Ghost Convolutions to reduce complexity [73].	**YOLO-NAS** achieved an outstanding 99.7% accuracy and 0.992 F1-score, beating traditional deep learning classifiers. **YOLOv8s + U-Net** hit 98.6% accuracy and 98.5% ROC-AUC score for complex brain structures. **YOLOv5x-GCB** improved mAP@0.5 to 0.931 for mixed intracranial hemorrhages compared to older generations.
**Thoracic and Pulmonary—Lung Lesions (CT and X-Ray)**	**YOLOv11:** Integrates Transformer-based attention layers and adaptive anchor-free mechanisms for small tumor detection [50]. **NSEC-YOLO:** Features an Adaptive Noise Suppression module to handle complex X-ray artifacts [57].	**YOLOv11** set a new benchmark with a 96.26% mAP and 95.76% IoU, outperforming YOLOv10 (95.23%) and YOLOv9 (95.70%) in locating lung cancer. **NSEC-YOLO** achieved an mAP@0.5 of 0.416 and 163 FPS, beating YOLOv7 and SSD in detecting small, overlapping lesions.
**Orthopedics—Bone Fracture Detection**	**Hybrid YOLO-NAS:** Advanced deep learning architecture applied to subtle hand bone fractures [82]. **YOLOv8:** Used for multi-class detection (severe vs. non-severe rib fractures) [46].	**Hybrid YOLO-NAS** hit a 97.85% mAP, vastly outperforming ResNet50, VGG19, and Vision Transformers. **YOLOv8** achieved a 0.972 mAP50 and 0.963 precision, reaching diagnostic performances comparable to expert thoracic surgeons.
**Dermatology and Wound Care-Pressure Ulcers and Skin Burns**	**YOLOv5s:** Customized to simultaneously detect and classify 4 distinct severity stages of pressure ulcers [106]. **YOLO-V5:** Applied to melanoma detection [87]. **YOLOv7:** Adapted for segmenting skin burn severity.	**YOLOv5s** achieved a 76.9% mAP50, overcoming the limitations of older binary CNN models that could only identify necrotic vs. non-necrotic tissue. **YOLO-V5** demonstrated high reliability with an mAP@0.5 of 0.935 for melanoma.
**Oncology (Mammography)—Breast Mass Detection**	**YOLOv5-CBAM:** Integrates a Convolutional Block Attention Module to focus on spatial and channel-wise features [75]. **YOLO-v8:** Anchor-free decoupled head for microcalcifications [47].	**YOLOv5-CBAM** improved the overall mAP to 0.887 (up from 0.785 in baseline YOLOv5) by aggressively filtering out false positives in dense breast tissue. **YOLO-v8** bypassed the slow region-proposals of Mask R-CNN, achieving faster global reasoning for tiny calcifications.
**Endocrinology—Thyroid Nodule Detection**	**YOLO-Thyroid:** Uses a Coordinate Attention (C2fA) module and class-weighted loss (CW-BCE) for data imbalance [62]. **Efficient-YOLO:** Uses EfficientNet backbone for lightweight feature extraction [99].	**YOLO-Thyroid** achieved 43.6% mAP0.5 and 58.2% recall, heavily boosting minority class detection. **Efficient-YOLO** hit 92.64% mAP while running at an ultra-fast 45.1 FPS.
**Hematology and Parasitology—Blood Cells and Malaria**	**SSW-YOLO:** Integrates SwinTransformer and SPD-Conv layers to detect densely packed, overlapping cells [110]. **YOLO-mp-3l:** Custom 3-layer architecture tailored for rural smartphone diagnosis [37].	**SSW-YOLO** achieved a 0.940 mAP@0.5, ensuring highly accurate real-time complete blood counts. **YOLO-mp-3l** achieved 93.99% mAP for *P. falciparum* malaria while requiring very low memory.
**Otolaryngology and Endoscopy—Airway Lesion Detection**	**SRE-YOLO:** Decoupled Super-Resolution branch trained but discarded during inference [79]. **BrYOLO-Mamba:** Incorporates State Space Models (SSM) for dynamic focal scanning [64].	**SRE-YOLO** boosted small lesion detection by 5% while maintaining a real-time 58.8 FPS. **BrYOLO-Mamba** achieved 83.29% accuracy, defeating heavy Vision Transformers on video streams.
**Gynecology—Cervical Precancer**	**YOLOv8xl + SAHI:** Uses Slicing Aided Hyper Inference to break down complex medical images [55].	**YOLOv8xl + SAHI** successfully identified ambiguous precancerous tissue that baseline models completely missed, improving AP by 1–2%.
**Emergency Medicine—Cricothyroidotomy Landmarks**	**YOLOv5s:** Adapted for real-time portable ultrasound screening [98].	**YOLOv5s** achieved a near-perfect AUC of 0.989 for identifying cricoid/thyroid cartilages to assist rapid emergency surgeries.

## Data Availability

No new data were created or analyzed in this study.

## Supplementary Materials

The following supporting information can be downloaded at: https://www.mdpi.com/article/10.3390/diagnostics16060935/s1, File S1: PRISMA 2020 Checklist.
